# Immune and inflammatory radiogenomics of radiotherapy-induced normal-tissue toxicity: genetic susceptibility, mechanisms, risk prediction, and clinical translation

**DOI:** 10.3389/fimmu.2026.1956614

**Published:** 2026-09-22

**Authors:** Shuo Zhang, YouTao Wu, Qiong Liu, Huan Yang, Xi Chen, YuZhe Song

**Affiliations:** 1 Department of Radiation Oncology, Radiation Oncology Key Laboratory of Sichuan Province, Sichuan Clinical Research Center for Cancer, Sichuan Cancer Hospital & Institute, Sichuan Cancer Center, University of Electronic Science and Technology of China, Chengdu, China; 2 Department of Cardiology, The First People’s Hospital of Neijiang, Sichuan, China; 3 Department of Endoscopy Center, Sichuan Clinical Research Center for Cancer, Sichuan Cancer Hospital & Institute, Sichuan Cancer Center, University of Electronic Science and Technology of China, Chengdu, China

**Keywords:** cytokines, fibrosis, germline variation, immune microenvironment, innate immunity, normal tissue, radiogenomics, radiotherapy-induced toxicity

## Abstract

Radiotherapy is indispensable for cancer control, yet acute and late normal-tissue toxicities remain major limits to treatment intensity and survivorship. Considerable interindividual variation persists after clinical and dosimetric factors are considered, motivating radiogenomic studies of inherited susceptibility. This review compares common and rare germline variation, association and sequencing designs, polygenic risk scores, and functional evidence through an immunological framework. Radiation-induced DNA damage and organelle stress release danger-associated signals and cytosolic DNA that engage pattern-recognition pathways, including cGAS-STING, and initiate endothelial and epithelial inflammatory programs. Cytokines, chemokines, inflammasomes, recruited myeloid cells, lymphocytes, senescent cells, and fibroblasts then determine whether injury resolves or progresses to chronic immune dysregulation and fibrosis. Germline variants may modify these responses, but pathway plausibility is not equivalent to variant-specific causality. The strongest evidence combines a replicated clinical association with dose adjustment, regulatory annotation, allele-specific perturbation, and validation in organ-relevant immune-stromal models. Integrated prediction should combine genomic susceptibility with normal-tissue imaging, dose-volume exposure, baseline inflammatory state, comorbidity, and systemic therapy. Germline associations with checkpoint-inhibitor toxicity provide a rationale for studying radio-immunotherapy, although radiation-specific genetic interactions remain unvalidated. No current common variant or polygenic score justifies reducing curative dose or target coverage. Near-term translation is more appropriately directed toward risk-adapted surveillance, supportive care, and genotype-enriched prevention trials. An immune-centered radiogenomic framework can connect inherited susceptibility to damage sensing, inflammatory amplification, resolution failure, and tissue remodeling while preserving the distinction between mechanistic evidence and clinical utility. Graphical summary: inherited susceptibility modifies the sequence from radiation-induced damage sensing and immune activation to resolution, repair, or chronic inflammation and fibrosis.

## 1 Introduction

Radiotherapy-induced normal-tissue toxicities include dermatitis, mucositis, pneumonitis, enteritis, genitourinary injury, cardiotoxicity, and late fibrosis, all of which can constrain treatment delivery or long-term quality of life (1–3). Traditional risk assessment relies on clinical factors, organ dose, irradiated volume, fractionation, concurrent systemic therapy, and comorbidity, but these variables do not fully explain marked interindividual variation among patients receiving comparable plans (1, 2). Host genomic susceptibility is therefore a plausible additional component rather than an alternative to established dosimetric predictors. Importantly, radiation injury is not only a cell-autonomous consequence of DNA damage. Irradiated epithelial, endothelial, stromal, and immune cells exchange danger signals, cytokines, chemokines, oxidants, and matrix-remodeling cues that can restore homeostasis or sustain organ dysfunction (4–7). Toxicity radiogenomics seeks to identify germline variants that modify this tissue immune response and to determine whether such information improves prediction beyond clinical and treatment factors (8, 9).

Toxicity must be defined as an organ-, grade-, and time-specific outcome. Acute mucositis during treatment, late rectal bleeding, breast induration two years after therapy, and patient-reported fatigue are not interchangeable endpoints. CTCAE, RTOG, LENT-SOMA, physician-rated scales, objective measurements, and patient-reported outcomes capture overlapping but distinct aspects of morbidity. Genetic effects may also vary over time: a variant that modifies the early inflammatory response may not predict late fibrosis. Harmonized definitions and repeated longitudinal assessment are therefore foundational requirements for both discovery and clinical prediction (8–10).

The term radiogenomics is used in two different ways across oncology. In toxicity research it refers to inherited variation and radiation response, whereas in imaging research it often denotes associations between radiomic features and tumor genomic characteristics. Both fields contribute to precision oncology, but they address different biological units and clinical endpoints. The primary focus here is inherited normal-tissue susceptibility, with a dedicated discussion of integration with imaging-based radiogenomics (Section 4.5). Tumor imaging and somatic genomic profiles may contribute complementary information about treatment benefit, but they are not substitutes for germline susceptibility data or direct evidence of normal-tissue toxicity (8–12).

A useful conceptual model is exposure-by-susceptibility interaction operating through an immune tissue-response continuum. Radiation dose and volume create the opportunity for injury; inherited biology may modify DNA and organelle damage sensing, endothelial activation, leukocyte recruitment, cytokine amplification, resolution, epithelial regeneration, and fibroblast remodeling. Cytosolic DNA sensing through cGAS-STING provides one bridge between genotoxic stress and inflammatory or senescence programs, whereas cytokine effects depend on dose, organ, cell source, timing, and receptor context (5, 13). This model explains why genomic markers should be interpreted alongside treatment planning data and longitudinal immune phenotypes rather than as fixed labels of radiosensitivity.

Clinical relevance also depends on absolute risk. The same relative genetic effect can have different consequences when the baseline incidence of an endpoint varies by organ dose, treatment technique, and population. A twofold relative risk may be important for a common moderate symptom yet produce a small absolute difference for a rare severe event. Reviews can therefore report absolute incidence whenever available and avoid translating an odds ratio directly into an individual probability. This distinction becomes critical when genomic information is proposed for consent discussions, surveillance, or treatment planning. A clinically interpretable estimate must combine the genetic component with the patient’s exposure and baseline clinical risk. Reporting both relative and absolute effects also makes results easier to compare across cohorts and helps distinguish a biologically interesting association from a result likely to change care. It also discourages deterministic language when individual uncertainty remains substantial (8–10).

### 1.1 Literature search and evidence classification

This narrative review was developed from targeted PubMed/MEDLINE-oriented searches, backward reference checking, and focused verification of all cited records. Search concepts combined radiotherapy or radiation injury with radiogenomics, germline variant, genome-wide association study, polygenic score, toxicity, fibrosis, mucositis, pneumonitis, cardiotoxicity, and normal-tissue injury. To strengthen immunological coverage, the search also included innate immunity, adaptive immunity, damage-associated molecular patterns, cGAS-STING, inflammasome, cytokine, chemokine, macrophage, lymphocyte, immune microenvironment, immune checkpoint inhibitor, senescence-associated secretory phenotype, and immune-mediated fibrosis. The original search through July 2026 was supplemented by targeted searches through 1 September 2026 covering rare-variant sequencing and aggregate association tests, germline-radiomic integration, HLA, *IL7*, and toxicity during radiotherapy combined with immune checkpoint inhibition. Further targeted searches addressed ancestry representation and transferability, genotype-by-dose interaction, chromatin-based variant-to-gene mapping, and stem/progenitor-cell reserve in normal-tissue injury. Priority was given to multicenter cohorts, genome-wide studies, meta-analyses, normal-tissue immune reviews, prospective toxicity studies, and experiments connecting a specific variant to a molecular or tissue phenotype.

Evidence was interpreted in four categories: association evidence, replication evidence, variant-specific functional evidence, and clinical-utility evidence. An association was not treated as replicated merely because a related pathway had been reported elsewhere, and pathway plausibility was not treated as proof of causality. Clinical utility required reproducible improvement beyond clinical and dosimetric variables plus support for a defined decision. Because the review was not a registered systematic review, it does not claim exhaustive coverage or provide a PRISMA-style study count. The resulting synthesis is therefore structured around evidentiary strength and translational readiness rather than a pooled effect estimate (**Figure 1**).

**Figure 1:**
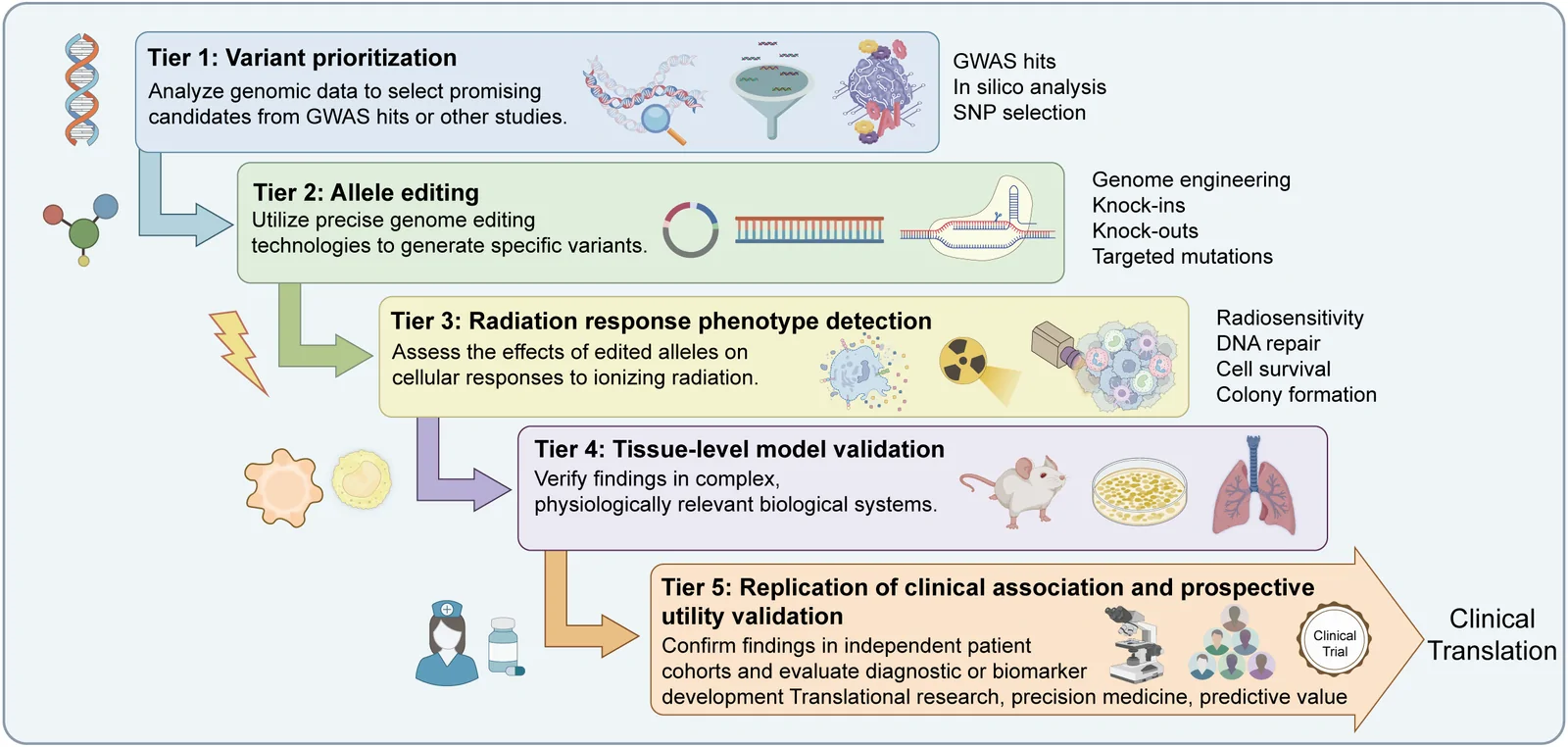
Evidence framework for interpreting radiogenomic associations with radiotherapy-induced normal-tissue toxicity.

## 2 Strategies for identifying genetic variants associated with radiotherapy toxicity

### 2.1 Candidate-gene association studies

Early toxicity radiogenomics used candidate-gene association analysis guided by radiobiological knowledge. Investigators selected genes involved in DNA damage response, cell-cycle control, oxidative stress, cytokine signaling, innate immune activation, and extracellular matrix remodeling. ATM, XRCC1, XRCC2, XRCC3, and LIG4 were frequently evaluated because their products participate in the recognition or repair of radiation-induced DNA lesions (14–16). TP53 rs1042522 and variants in TGFB1, TGFBR1, TNF, and antioxidant pathways have also been associated with acute mucosal, skin, genitourinary, or fibrotic endpoints in particular cohorts (15, 17, 18). Immune-pathway candidacy should be specified at the level of allele, cell type, radiation context, and endpoint: a cytokine gene is not a toxicity marker merely because its product participates in inflammation. Evidence is most informative when the tested allele has a prespecified functional rationale, analyses control for dose-volume exposure and population structure, and the result is replicated in an independent cohort.

Reproducibility has been the central limitation of candidate-gene studies. Several analyses failed to confirm previously reported associations involving XRCC1, XRCC2, XRCC3, or TGFB1-related variants (16, 18). Disagreement can arise from small samples, selective reporting, multiple testing, population stratification, treatment technique, fractionation, organ dose, concurrent chemotherapy, inconsistent covariate adjustment, and non-equivalent definitions of toxicity (10, 19). A study of acute grade 2 mucositis during chemoradiotherapy and a study of patient-reported xerostomia two years after treatment do not measure the same phenotype, even when both arise in head and neck cancer. Similarly, an association observed after two-dimensional radiotherapy may not reproduce after intensity-modulated or image-guided treatment because the distribution of normal-tissue exposure has changed. These sources of heterogeneity reduce statistical power and can produce apparently contradictory estimates without necessarily disproving the underlying biology.

Despite these limitations, candidate-gene studies established a useful conceptual link between inherited variation and interindividual radiation response. Their main value was to prioritize pathways and define testable hypotheses, not to provide stand-alone clinical tests. Subsequent GWAS of acute oral mucositis, breast toxicity, urinary toxicity, and cross-cancer acute toxicity identified loci outside the most frequently studied candidate genes, illustrating the limitations of relying only on prior biological assumptions (19–27). Candidate evidence can therefore be integrated with unbiased discovery, replication, fine mapping, expression quantitative trait locus analysis, and functional experiments (**Figure 2**). A locus becomes more credible when genetic association, relevant normal-tissue expression, regulatory annotation, and radiation-dependent cellular phenotype converge on the same mechanism.

**Figure 2:**
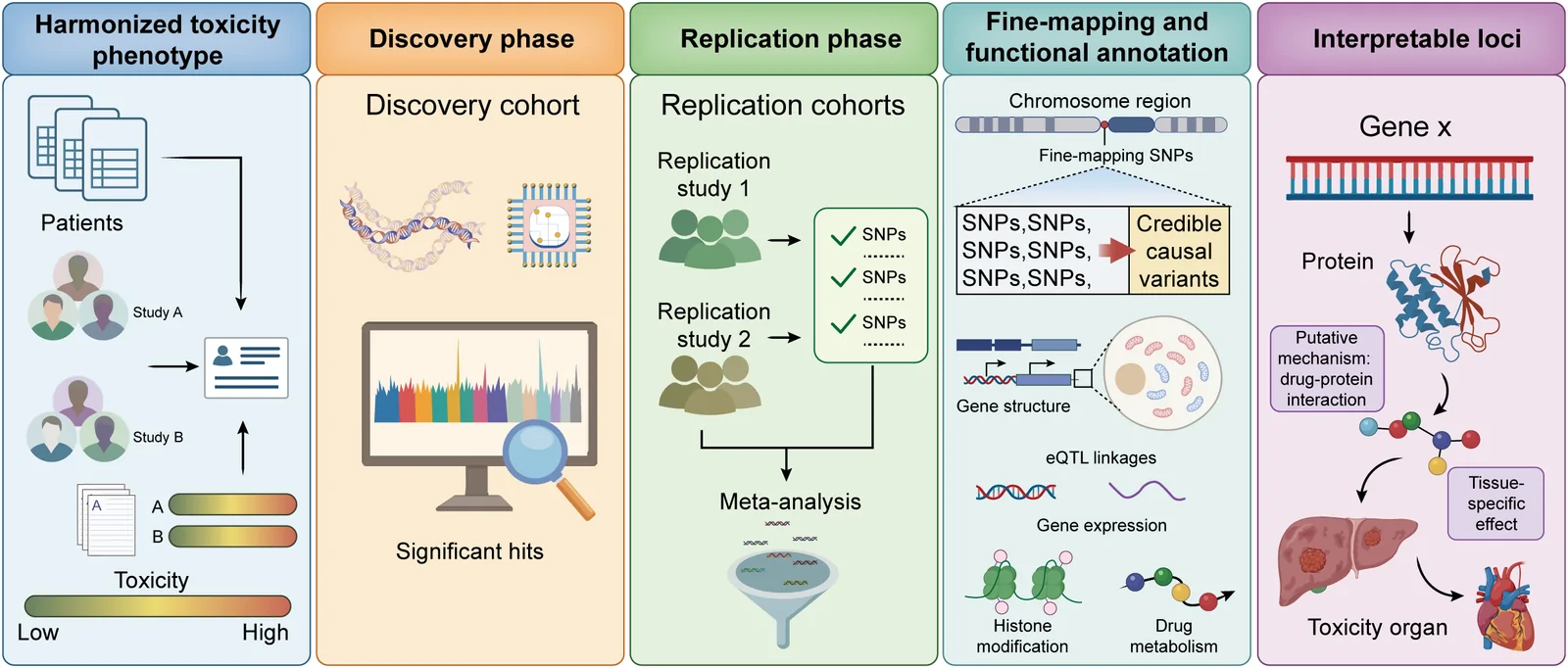
Discovery, replication, and interpretation pipeline for toxicity radiogenomics.

Candidate-gene results are especially vulnerable to analytic flexibility. Investigators may test several genetic models, toxicity cutoffs, follow-up windows, and subgroups, creating more effective comparisons than are visible in the final report. Prospective analysis plans, correction for multiple testing, registration of endpoints, and reporting of all evaluated variants reduce this risk. Effect estimates can be accompanied by confidence intervals and allele frequencies, allowing readers to distinguish a precise modest association from an unstable large odds ratio based on few events (10, 14–19).

Dose-volume adjustment deserves particular attention because genotype may appear associated with toxicity when carriers happened to receive higher organ doses. Adjustment for dose as a main effect does not, however, test whether genotype modifies the dose-response relationship; that question requires an explicit interaction model. Analyses can report both main effects and prespecified genotype-by-dose interactions when sample size permits. Standardized extraction of dose-volume histograms, spatial dose maps, and fractionation-adjusted metrics can make cross-cohort replication more meaningful (21).

### 2.2 Genome-wide association studies and cross-cohort meta-analysis

Genome-wide association studies scan common germline variation without restricting the analysis to predefined pathways (24). In toxicity research, this approach can reveal susceptibility loci that would not be selected from conventional radiobiological models. A GWAS of radiation-induced acute oral mucositis identified loci and enriched pathways related to telomere regulation, including an association near TNKS (19). Studies of prostate cancer toxicity identified signals related to late hematuria and biological hypotheses involving the renin-angiotensin system (25). These observations demonstrate the discovery potential of GWAS, but genome-wide significance is not equivalent to causality. Index SNPs may tag nearby functional variants through linkage disequilibrium, and the nearest gene is not necessarily the causal gene. Credible-set construction, colocalization, chromatin annotation, and perturbation experiments are needed before a locus is assigned to a specific molecular pathway.

Large prospective cohorts have improved phenotype quality and statistical power. In the REQUITE breast cancer cohort, genome-wide analyses evaluated toxicity two years after radiotherapy and identified variants associated with grade 2 or greater breast edema and induration (26). The study also failed to reproduce some earlier candidate associations, which is scientifically informative because it constrains the set of variants likely to be robust. Reported heritability estimates indicate that common genetic variation may explain a proportion of toxicity liability, but estimates depend on endpoint definition, sample composition, follow-up, and statistical model (**Table 1**). Heritability does not imply that a clinically accurate predictor already exists: a trait can be partly heritable while the available cohort remains too small to estimate thousands of variant effects precisely.

**Table 1:** Representative human radiogenomic studies and their evidentiary status.

Study/setting	Toxicity endpoint	Principal signal	Exposure/phenotype context	Evidence status
(19) Oral-mucositis GWAS	Acute oral mucositis	Signals involving telomere regulation, including a locus near TNKS	Organ-specific acute phenotype in a genome-wide discovery setting	Association signal; external replication and variant-level functional work remain necessary
(14) Extreme-toxicity case	Severe acute toxicity	Rare variants in XRCC1, XRCC3, and LIG4	Individual case with toxicity judged disproportionate to the delivered treatment	Hypothesis-generating rare-variant evidence; no population-level effect estimate
(18) Prostate candidate-gene study	Prostate radiotoxicity	Associations involving TGFB1 and TGFBR1	Candidate-pathway genotyping with clinically scored toxicity	Association evidence; reproducibility across cohorts remains limited
(20) Two-stage nasopharyngeal-cancer GWAS	Acute toxicity after radiotherapy	Novel susceptibility loci	Two-stage design with site-specific acute endpoints	Discovery/replication framework; causal genes and mechanisms remain unresolved
(21) Prostate spatial-dose/genetic study	Rectal toxicity	Genetic information integrated with the posterior rectal dose surface	Spatial exposure modeling rather than summary dose alone	Demonstrates dose–genotype integration; locus-level utility awaits validation
(22, 23) Head-and-neck GWAS and meta-GWAS	Mucositis, dysphagia, xerostomia, and composite acute burden	Suggestive loci and modest SNP heritability; polygenic scores explained less than 3% of variance	Multicohort discovery and replication with heterogeneous acute phenotypes	No genome-wide significant replicated SNP in the meta-GWAS; supports a polygenic architecture
(26) REQUITE breast GWAS	Two-year breast edema, induration, nipple retraction, and arm lymphedema	Eight genome-wide significant SNPs across distinct endpoints	1, 640 patients of European ancestry; CTCAE v4 scoring with clinical and treatment adjustment	Earlier SNPs were not replicated; independent validation remains necessary
(27) Cross-cancer meta-GWAS	Standardized acute toxicity across cancer sites	Measurable SNP heritability and a suggestive signal near DPPA4	Nineteen cohorts comprising 12, 042 patients	Supports shared susceptibility while retaining substantial organ- and phenotype-specific heterogeneity
(35) KDM3B–LOX functional study	Postradiation prostate stromal fibrosis	rs17599026–KDM3B–m6A–LOX–collagen regulatory chain	Prime-edited cells and genotype-informed mouse experiments	Strong variant-specific functional evidence; clinical replication and utility remain pending

International collaboration enables pooled analysis of summary statistics and harmonized individual-level data. A meta-GWAS involving 19 cohorts and 12, 042 patients across prostate, head and neck, breast, and lung cancer evaluated a standardized acute-toxicity phenotype and estimated a measurable SNP-heritability component (27). The strongest suggestive signal was located near DPPA4, a gene with potential relevance to cellular plasticity and tissue repair, although suggestive association requires further replication. Cross-cancer analysis can identify shared host-response mechanisms, but it can also obscure organ-specific effects if mucosal, pulmonary, breast, and urinary outcomes are compressed into a single score. Cancer-site-stratified estimates, heterogeneity tests, and sensitivity analyses are therefore essential. By contrast, germline associations with tumor response, such as CACNA2D3 findings in nasopharyngeal carcinoma (28), concern therapeutic efficacy rather than normal-tissue toxicity and should not be presented as direct toxicity evidence.

Phenotype harmonization should not mean forcing biologically distinct outcomes into a single category. Core outcome sets can define minimum variables while preserving organ-specific detail. Useful fields include baseline symptoms, maximum acute grade, persistent grade, time to onset, recovery, late grade, objective function, and patient-reported burden. Immune characterization can add baseline autoimmune disease, infection, corticosteroid or anti-inflammatory exposure, circulating leukocyte states, and longitudinal cytokine measurements when these variables are mechanistically relevant. A shared data dictionary can also capture surgery, systemic therapy, medications, comorbidity, smoking, microbiome-modifying treatment, and technical radiotherapy parameters (20–27).

Ancestry diversity is both a scientific and an equity requirement. In a historical review of 174 toxicity-related radiogenomic studies, only 23 included more than one racial or ancestral group, and none recruited in Africa; recruitment geography should nevertheless be distinguished from participants’ genetic ancestry (8). Non-European evidence includes an early prostate-toxicity GWAS in 79 African-American men and the oral-mucositis GWAS in 1, 467 Chinese patients with nasopharyngeal carcinoma (19, 29). These examples do not offset the European-ancestry predominance highlighted by the Radiogenomics Consortium in 2024 (30). Large, prospectively phenotyped cohorts remain particularly needed for populations of African or South Asian ancestry, Indigenous populations, and diverse populations in Latin America, including admixed groups. Representation must also extend across irradiated organs and treatment settings: evidence from one ancestry-associated cancer and treatment context cannot establish transferability to other organs or regimens (30, 31).

Principal-component adjustment addresses broad population structure but cannot ensure that an association is free of ancestry-related confounding. In admixed populations, local ancestry at a locus may differ from genome-wide ancestry; fine-scale structure, relatedness, and ancestry-dependent linkage disequilibrium or imputation accuracy can also remain relevant (32). Moreover, ancestry may correlate with recruiting center, dose distribution, systemic treatment, baseline morbidity, access to care, or completeness of toxicity follow-up. Such correlations can produce apparent genetic differences when exposure or ascertainment is incompletely measured (31). Analyses therefore require harmonized dosimetry and endpoints, ancestry-appropriate quality control and reference panels, and evaluation of center and treatment effects alongside genetic structure. Relatedness-aware models and, where appropriate, local-ancestry analyses should complement ancestry-stratified estimates and tests of heterogeneity (32, 33). Neither additional principal components nor more complex models can resolve complete confounding between ancestry and treatment center when comparable exposure groups are absent.

Direct transfer of European-derived associations presents a separate problem even when the discovery result is valid. A lead SNP may tag a causal allele less effectively, or with a different linkage-disequilibrium phase, in another population; allele-frequency differences and imperfect imputation can further reduce predictive information. Consequently, a marker’s estimated effect need not transport even if the underlying biological effect is shared (32, 34). In the prostate-cancer meta-analysis of 3, 871 European-ancestry men, evaluation in 962 Japanese men provided partial support for urinary-toxicity signals, with similar effects for some variants in photon-treated patients; it did not establish universal transferability across variants or treatment modalities (33). This distinction also prevents an underpowered failure to replicate from being interpreted automatically as a biological ancestry difference.

For clinical prediction, differences in linkage disequilibrium, allele frequency, baseline event rates, and treatment exposure may impair both discrimination and calibration of a European-derived polygenic score (34). An overestimated toxicity risk could prompt unnecessary treatment compromise, whereas an underestimated risk could reduce appropriate surveillance. Before clinical use, models require validation of absolute risk, calibration, uncertainty, and decision benefit in the intended population and treatment setting. Recruitment of underrepresented groups, suitably powered multi-ancestry discovery, and population-specific recalibration are therefore complementary requirements; self-reported race or ethnicity should not substitute for genetic ancestry or for measurement of social and treatment-related determinants (30–32, 34).

### 2.3 Phenotype definition, exposure modeling, and reproducibility

A radiogenomic phenotype begins with a clear clinical question. Investigators can specify whether the goal is etiologic discovery, absolute-risk prediction, identification of extreme radiosensitivity, or selection of a preventive intervention. These goals require different designs. Etiologic studies may tolerate a broad mechanistic endpoint if exposure and confounding are controlled, whereas a clinical prediction model must estimate risk at a defined decision time using variables available at that time. Mixing discovery and prediction objectives often leads to exaggerated claims: a statistically significant association can illuminate biology without providing useful individual prediction (21, 22, 26, 27).

Baseline assessment is essential because many symptoms attributed to radiation are present before treatment. Xerostomia, urinary frequency, bowel dysfunction, pain, fatigue, and skin changes may reflect the tumor, prior surgery, age, medication, or comorbidity. Analyses based only on post-treatment grade can therefore misclassify prevalent symptoms as incident toxicity. Change from baseline, time to clinically meaningful deterioration, and persistent burden are often more interpretable. Patient-reported outcomes can detect effects missed by clinician grading, but validated instruments and prespecified thresholds are needed (21, 22, 26, 27).

Radiation exposure is multidimensional. Mean organ dose, maximum dose, dose-volume histogram points, spatial dose distribution, fraction size, overall treatment time, and irradiated substructure may each be relevant. A single prescribed dose is rarely an adequate surrogate for normal-tissue exposure. Spatial analyses of the rectum illustrate how genetic information can be evaluated together with the location of dose rather than with a summary measure alone (21). Similar approaches may be important for salivary glands, lung, heart, bowel, and bladder, where function depends on nonuniform anatomical subregions.

A genetic main effect and a genotype-by-dose interaction describe different aspects of susceptibility. A main effect represents a genotype-associated difference in toxicity at a defined reference dose, whereas interaction indicates that the dose-response relationship changes with genotype on a specified analytical scale. Reporting a genetic association after adjustment for dose addresses the former question and does not establish the latter. For a binary toxicity endpoint, one illustrative model is (36):


$$\text{logit}\left[\text{P}\left(\text{Y}=1\right)\right]=\alpha +\beta_{\text{G}}\text{G}+\beta_{\text{D}}{\text{D}}_{\text{c}}+\beta_{\text{GD}}\left(\text{G}\times {\text{D}}_{\text{c}}\right)+\gamma^{\text{T}}\text{C}$$

Here, G is allele dosage, D_c_ is an organ-dose metric centered at a clinically relevant reference dose, and C contains prespecified clinical covariates. The reference dose should lie within the dose range adequately represented in each genotype group. The coefficient *β*_G_ estimates a genetic contrast at that dose; this “baseline” risk is distinct from pretreatment symptoms or risk without irradiation. When *β*_GD_ = 0, the genetic effect is constant on the log-odds scale. When *β*_GD_ differs from zero, the log-odds dose-response slope is *β*_D_ + *β*_GD_G. Because the inverse-logit transformation is nonlinear, absolute-risk curves can have different slopes even without interaction on the log-odds scale. Likewise, a genotype-associated shift in the dose producing a given toxicity probability does not alone demonstrate slope modification. The effect scale should therefore be specified, with genotype-specific absolute-risk curves and confidence intervals reported for clinical interpretation (36).

Main effects can be estimated in an exposure-adjusted model, but slope modification requires a direct likelihood-ratio or Wald test of the interaction term while retaining both genetic and dose main effects. A significant dose association in one genotype subgroup and a nonsignificant association in another is insufficient evidence of interaction. For nonlinear responses, genotype-by-spline terms can be tested jointly; ordinal or time-to-event formulations may be preferable for toxicity grade or latency. Confounder adjustment for interaction also deserves care: relevant covariate-by-genotype and covariate-by-dose terms may be needed, because adjustment for covariate main effects alone can leave an interaction estimate confounded (37). Dose metrics and tests should be prespecified, with multiplicity addressed across variants, exposure metrics, and endpoints. Restricting interaction testing to SNPs with significant marginal effects may miss variants whose effects emerge only at particular doses.

Dose-modifying normal-tissue complication probability models provide a related approach. Tucker et al. incorporated SNP-dependent dose-modifying factors into a Lyman model for pneumonitis in 141 patients with non-small cell lung cancer, using mean lung dose as the exposure (38). This was an exploratory example of combining genetic and dosimetric information; the assumed dose rescaling does not by itself validate an unrestricted genotype-specific slope or a clinical dose-reduction rule. Reliable interaction estimates require sufficient events and overlapping dose distributions within genotype groups, replication across comparable fractionation schedules, and validation using actual normal-organ exposure. Narrow dose ranges, rare genotype groups, and correlated dose-volume metrics can make interaction estimates unstable even when the main genetic effect is estimable. Any proposed genotype-guided dose constraint should subsequently demonstrate clinical utility while preserving tumor control.

Treatment interactions can be planned rather than explored opportunistically. Concurrent chemotherapy, immune checkpoint inhibition, hormonal therapy, targeted therapy, surgery, and supportive medication can modify tissue response (39, 40). Checkpoint blockade is particularly relevant because antitumor immune activation and normal-tissue inflammation can share cytokine, endothelial, and lymphocyte pathways, while the combined risk still depends on organ dose, sequence, baseline autoimmune status, smoking, and pulmonary reserve. If a germline variant acts through the same immune pathway perturbed by treatment, its effect may be exposure dependent; however, a germline association with drug-associated immune toxicity does not by itself establish a genotype-by-radiation interaction.

Population-structure assessment should combine genotype quality checks, ancestry principal components, relatedness assessment, and appropriate imputation reference panels, with additional evaluation of residual confounding as discussed above. Batch effects can create false associations if cases and controls are genotyped or processed differently. Variant call rate, Hardy-Weinberg equilibrium, allele frequency, imputation quality, and sample contamination can be reported. These procedures are routine in contemporary GWAS but are particularly important in radiotherapy cohorts, which are often assembled across institutions and treatment eras (21, 22, 26, 27).

Replication can use the same allele orientation, genetic model, endpoint, time window, and covariate structure whenever possible. A nominal association with a related but nonidentical endpoint is supportive but not a strict replication. Meta-analysis can report heterogeneity and inspect whether one cohort drives the result. Random-effects estimates may be appropriate when genuine clinical differences exist, although they do not solve incompatible phenotype definitions. Sharing harmonized summary statistics enables independent reanalysis and future polygenic modeling (21, 22, 26, 27).

Statistical significance can be separated from clinical importance. A variant with a small effect can be biologically informative yet contribute little to absolute-risk prediction. Conversely, a large estimated odds ratio may reflect sparse events or winner’s curse and shrink substantially in replication. Reporting event counts by genotype, absolute risks, confidence intervals, and incremental model performance helps readers evaluate importance. Claims of patient stratification can be reserved for results that meaningfully separate calibrated risk groups in independent data (21, 22, 26, 27).

Open and reproducible workflows can accelerate the field. Common data models, versioned phenotype definitions, analysis code, and durable documentation allow cohorts to contribute without releasing identifiable genomic data. Federated or summary-statistic approaches may support international collaboration where data transfer is restricted. Reproducibility also depends on recording genome build, variant identifiers, allele coding, imputation panel, software versions, and quality-control thresholds. These details prevent avoidable strand and mapping errors when results are combined (21, 22, 26, 27).

### 2.4 Endpoint-specific considerations

Acute and late endpoints can be analyzed separately unless a composite has been validated. Acute toxicity often reflects epithelial depletion, endothelial activation, edema, innate immune recruitment, and cytokine signaling during or shortly after treatment. Late toxicity more often involves persistent macrophage and lymphocyte states, unresolved cytokine signaling, vascular injury, senescence, atrophy, and fibrosis (4, 6, 7, 41, 42). The same immune pathway may contribute to both phases, but its cellular source and effect direction can change over time. A composite that gives equal weight to acute mucositis and late fibrosis may therefore obscure distinct genetic and immunological architectures (1, 2, 22, 26, 27).

Organ-specific outcomes require anatomical precision. Radiation pneumonitis depends on lung exposure and systemic context, whereas central-airway injury reflects high dose to a different structure (1, 2). Rectal bleeding, urinary frequency, hematuria, and erectile dysfunction are separate pelvic endpoints. Breast edema, induration, pain, and cosmetic change likewise should not be collapsed without justification (26). Clear anatomical definitions improve biological interpretation and the transfer of findings to treatment planning.

Severity thresholds influence both power and meaning. Dichotomizing grade 0–1 versus grade 2 or greater is common but discards ordering and may combine moderate, severe, and life-threatening events. Ordinal, continuous, or time-to-event models can use more information when assumptions are appropriate. Sensitivity analyses across thresholds help determine whether an association reflects general symptom burden or only the most severe tail (1, 2, 22, 26, 27).

Follow-up must be adequate for the endpoint. Patients without late toxicity at one year cannot be considered long-term controls for complications that emerge over several years. Death, recurrence, salvage treatment, and loss to follow-up can create competing risks or informative missingness. Studies can report follow-up distributions and use methods consistent with the clinical process. Survivorship cohorts can contribute valuable late outcomes but may exclude patients with early mortality or severe disease (1, 2, 22, 26, 27).

External events can change measured toxicity after radiotherapy. Infection, trauma, new medication, additional surgery, recurrence, and reirradiation may mimic or exacerbate the original injury. Adjudication rules and source documentation help distinguish treatment-related events. Where attribution is uncertain, analyses can compare all-cause organ dysfunction with more specific adjudicated endpoints. Transparent uncertainty is preferable to excluding complex patients without explanation (1, 2, 22, 26, 27).

### 2.5 Common SNPs and rare functional variants: complementary designs and clinical implications

Allele frequency, molecular consequence, and clinical pathogenicity are distinct properties. For this comparison, common variants have a minor allele frequency (MAF) of at least 5%, low-frequency variants 1% to less than 5%, and rare variants less than 1%; individual studies may use different thresholds (43). Rare variants include coding, splice-altering, regulatory, and structural changes. Rarity or predicted functional disruption alone does not establish pathogenicity, which requires disease- and inheritance-specific interpretation (44).

Common-SNP studies generally require large, well-phenotyped radiotherapy cohorts because individual effects are modest. Genotyping arrays with imputation support genome-wide discovery; adjusted single-variant regression, mixed models, or time-to-event analyses account for ancestry, relatedness, organ dose, and clinical covariates. Genome-wide multiplicity control, conventionally P < 5 × 10^–8^ for common-variant GWAS, and independent replication limit false discovery. The REQUITE breast study and cross-cancer meta-GWAS illustrate this population-based strategy (26, 27). Their outputs are association estimates and potential inputs to toxicity-specific polygenic scores, not diagnoses of a radiosensitivity syndrome.

Rare-variant discovery instead benefits from sequencing patients with toxicity disproportionate to documented exposure, informative families, and adequately exposed controls. Targeted panels, exome sequencing, and genome sequencing offer progressively broader coverage, although exomes incompletely capture regulatory and structural variation. Comparable sequencing and variant-calling pipelines, ancestry-aware frequency filtering, and confirmation of prioritized calls are essential (43). The extreme-toxicity case involving *XRCC1*, *XRCC3*, and *LIG4* illustrates hypothesis generation rather than proof of a causal allele (14). Population databases help assess rarity but cannot replace radiotherapy-exposed controls when estimating toxicity risk.

Sparse carrier counts make conventional single-variant analyses unstable. Gene- or region-based burden tests pool qualifying alleles and are most efficient when effects are predominantly in the same direction. The sequence kernel association test (SKAT) accommodates heterogeneous directions and magnitudes, whereas SKAT-O adaptively combines burden and variance-component approaches (45, 46). Qualifying-variant masks, frequency thresholds, endpoints, and covariates should be prespecified, with correction across genes and masks. A significant aggregate test does not establish that every included allele is damaging. Replication should preserve the gene definition and mask; segregation, when informative, and allele-specific functional assays then evaluate individual candidates.

Clinical interpretation also differs. Common alleles generally modify an endpoint-specific probability, whereas some rare biallelic pathogenic variants cause recognized radiosensitivity disorders. These categories cannot be generalized across genes or zygosity (47). In a retrospective breast-radiotherapy cohort of 91 *ATM*-variant carriers, including 23 with pathogenic variants, no excess toxicity associated with pathogenic status was detected over a median 32-month follow-up; the small cohort and limited follow-up restrict conclusions about uncommon or very late events (48). Thus, heterozygous cancer predisposition does not automatically imply severe tissue radiosensitivity. Clinically established syndromes may warrant specialist genetic evaluation and individualized planning, whereas a variant of uncertain significance should not direct treatment modification (44, 47). Absolute penetrance requires appropriately ascertained, exposed cohorts rather than extrapolation from selected severe cases. **Table 2** summarizes the complementary designs and clinical implications.

**Table 2:** Comparison of common-SNP and rare-variant approaches to normal-tissue toxicity.

Dimension	Common SNPs	Rare putatively functional variants
Frequency and meaning	Usually MAF ≥5%; functional and nonfunctional alleles.	Operationally MAF <1%; rarity does not establish pathogenicity.
Discovery design	Large exposed cohorts; arrays and imputation; multicohort GWAS (26, 27).	Exposure-matched extremes or families; panels, exomes, or genomes; broader-cohort confirmation (43).
Primary analysis	Adjusted single-variant models; genome-wide multiplicity control; LD-aware PRS.	Prespecified masks; burden, SKAT, or SKAT-O; correction across genes and masks (45, 46).
Validation	Same allele, endpoint, and exposure context; fine mapping; independent model evaluation.	Same gene and mask; confirmed calls; informative segregation and allele-specific functional evidence.
Risk interpretation	Usually incremental risk; calibrated, organ- and time-specific absolute estimates.	Gene- and zygosity-dependent effects; selected cases cannot determine population penetrance.
Clinical implications	Investigational risk stratification; prospective utility evidence required.	Established syndromes may affect specialist planning; heterozygosity is not uniformly radiosensitive (47, 48); VUS is not actionable (44).

GWAS, genome-wide association study; LD, linkage disequilibrium; MAF, minor allele frequency; PRS, polygenic risk score; SKAT, sequence kernel association test; SKAT-O, optimal combined SKAT and burden test; SNP, single-nucleotide polymorphism; VUS, variant of uncertain significance. Frequency categories are operational and do not replace clinical variant classification.

## 3 Biological pathways linking germline variation to normal-tissue toxicity

### 3.1 DNA damage response and repair

Ionizing radiation creates DNA base damage, single-strand breaks, double-strand breaks, and clustered lesions in exposed normal and tumor cells (49, 50). Double-strand breaks are particularly consequential because inaccurate or incomplete repair can lead to cell death, chromosomal instability, senescence, or persistent inflammatory signaling. ATM, PRKDC (encoding DNA-PKcs), LIG4, and XRCC family genes participate in damage sensing and repair (14, 51). Rare biallelic loss-of-function disorders and knockout models demonstrate that severe disruption of these pathways can produce marked radiosensitivity. Such evidence establishes biological necessity but should not be conflated with the modest effects of common polymorphisms in the general population. The relevant radiogenomic question is whether a specific germline allele changes normal-tissue repair sufficiently to influence a defined clinical endpoint after accounting for the delivered dose.

Non-homologous end joining and homologous recombination are major pathways for repair of double-strand breaks (52). DNA-PKcs is central to non-homologous end joining, whereas RAD51 supports homologous recombination. Pharmacologic inhibition of ATM, DNA-PKcs, or RAD51 can increase tumor radiosensitivity, but these experiments do not directly demonstrate that common germline variants cause clinical toxicity (51, 53). They instead show that reducing repair capacity can narrow the therapeutic window when normal tissues are also exposed. An extreme-toxicity case in a patient carrying rare variants in XRCC1, XRCC3, and LIG4 provides a clinically relevant example of how unusual combinations of repair defects may contribute to radiosensitivity (14). Case reports, however, cannot estimate population risk or establish which variant is responsible. Functional assays and comparison with variant frequencies in matched populations are required.

Persistent or misrepaired DNA damage can initiate delayed toxicity through genomic instability, mitochondrial dysfunction, apoptosis, and cellular senescence (54–56). DNA fragments that accumulate in the cytosol can engage cGAS-STING and downstream interferon or inflammatory signaling, while senescent epithelial, endothelial, or stromal cells may secrete cytokines and matrix-remodeling factors (13). The consequence is not predetermined: transient sensing may support immune clearance and repair, whereas persistent signaling can maintain inflammatory recruitment and fibrosis. Germline variants in repair or sensing pathways should therefore be tested for allele-dependent, radiation-dependent immune outputs rather than inferred from pathway membership alone.

Functional annotation can prioritize variants within a GWAS locus. Colocalization with expression or splicing quantitative trait loci, allele-specific chromatin accessibility, promoter-enhancer interaction, and tissue-specific expression can narrow the plausible target gene. Because many regulatory effects are context dependent, annotation from unirradiated blood may miss responses that occur only after DNA damage in lung, mucosa, bladder, breast, skin, or intestinal tissue. Time-resolved profiling before and after irradiation is therefore more informative than static annotation alone (14, 49–57).

The distinction developed in Section 2.5 is particularly important for DNA repair genes: evidence from a biallelic loss-of-function disorder or a knockout model cannot be transferred directly to a heterozygous carrier or a common regulatory allele. Functional validation should therefore reproduce the actual allele, zygosity, and residual repair capacity, and distinguish effects on radiation response from cancer predisposition alone (14, 47, 48).

### 3.2 Inflammatory and immune response pathways

#### 3.2.1 Damage sensing and innate immune activation

Ionizing radiation generates immunological signals in normal tissue through damaged DNA, micronuclei, mitochondrial stress, oxidized molecules, extracellular ATP, HMGB1, and other danger-associated molecular patterns. Pattern-recognition receptors in irradiated parenchymal cells, endothelium, resident macrophages, and dendritic cells translate these cues into NF-kB-, interferon-, and inflammasome-associated programs (4, 6, 13). cGAS-STING is a prominent bridge between cytosolic DNA, type I interferon signaling, senescence, and inflammation, but the magnitude and duration of activation depend on dose, fractionation, nucleases, cell type, and tissue context (13). A radiogenomic hypothesis is strongest when a germline allele changes this stimulus-response relationship in an organ-relevant normal-cell system and the same allele associates with a defined clinical toxicity.

#### 3.2.2 Cytokine, chemokine, and inflammasome networks

Cytokines and chemokines form a dynamic communication network rather than a linear cascade. TNF, IL-1 family members, IL-6, type I interferons, TGF-beta, CXCL and CCL chemokines, and adhesion molecules influence vascular permeability, leukocyte trafficking, epithelial survival, macrophage state, and fibroblast activation (5–7, 58–62). Their effects can be protective or injurious according to concentration, cellular source, receptor expression, and timing; an early signal that supports antimicrobial defense or regeneration may become pathogenic when sustained. Inflammasome activation can further couple cellular damage to IL-1 family signaling. Consequently, one post-treatment cytokine measurement cannot establish mediation, and candidate variants require genotype-stratified longitudinal measurements plus variant-specific functional evidence.

#### 3.2.3 Immune-cell recruitment, adaptive immunity, and resolution

Radiation recruits neutrophils, monocytes, macrophages, and lymphocytes into injured normal tissues. Myeloid cells may remove debris and support repair, yet persistent oxidant, protease, and profibrotic programs can amplify parenchymal injury. T lymphocytes are recurrent in irradiated normal tissue, although their effects vary across organ, subset, and disease phase; regulatory, effector, and tissue-resident states cannot be reduced to a single beneficial or harmful category (4, 41). Resolution is therefore an active immunological process involving cessation of recruitment, clearance of damaged cells, restoration of barriers, and re-establishment of stromal homeostasis. Germline susceptibility may act by shifting this balance rather than simply increasing the peak inflammatory response (**Figure 3**).

**Figure 3:**
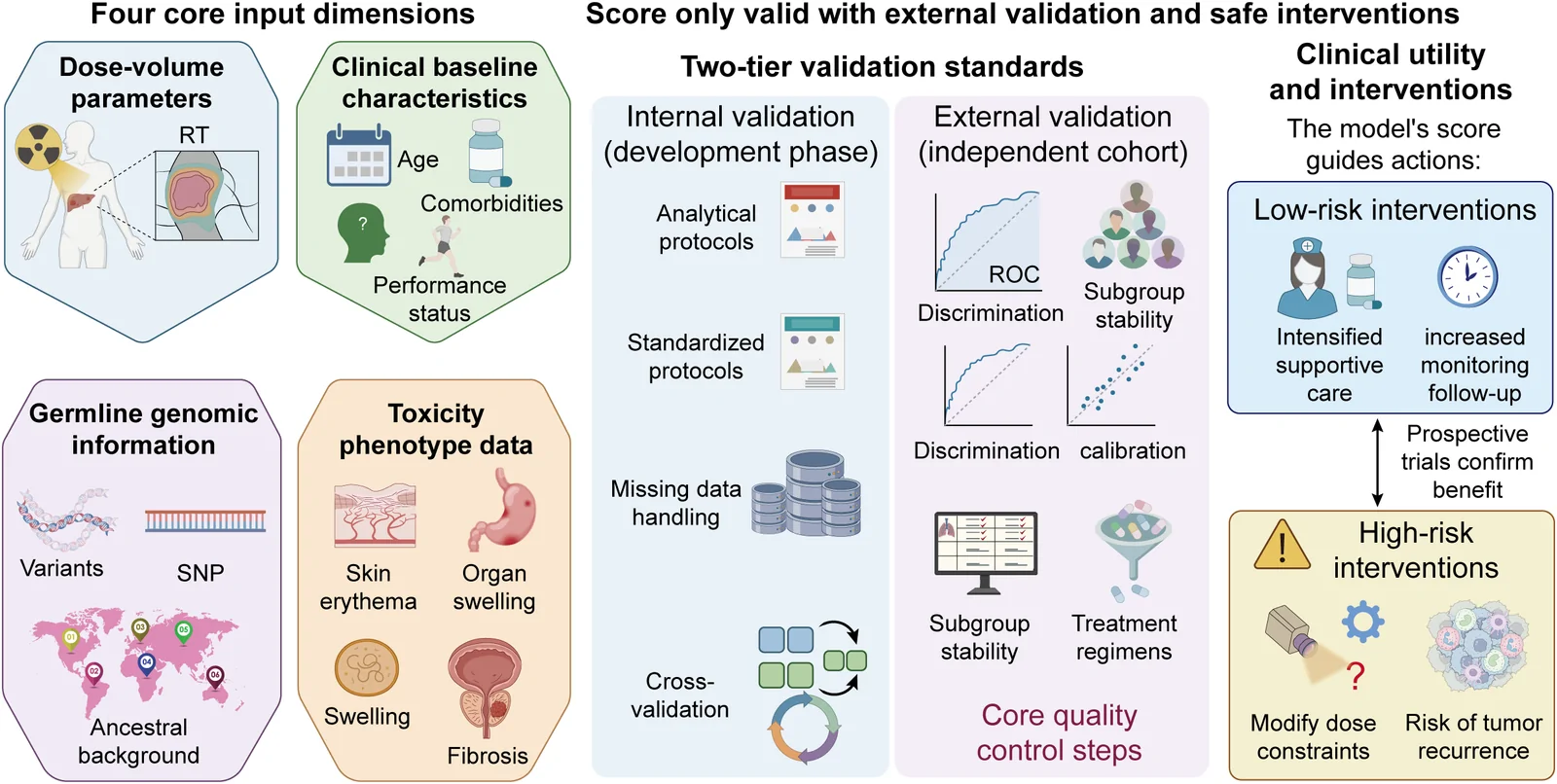
Integrated pathways linking radiation exposure and inherited susceptibility to acute and late normal-tissue injury.

#### 3.2.4 Germline susceptibility to toxicity from radiotherapy combined with immune checkpoint inhibitors

Radiotherapy combined with immune checkpoint inhibitors (ICIs) introduces an exposure context in which tissue injury and loss of immune tolerance may overlap. In the exploratory PACIFIC analysis, grade ≥2 pneumonitis or radiation pneumonitis occurred in 19.8% of durvalumab-treated patients and 14.1% of placebo-treated patients after concurrent chemoradiotherapy; grade ≥3 rates were 4.6% and 4.7%, respectively (63). These findings establish a clinically relevant combined-treatment phenotype, but neither demonstrate a genetic mechanism nor imply that severe toxicity increases uniformly with ICI exposure. Attribution to radiation versus immune-mediated injury was uncertain, and a pooled pneumonitis endpoint was used.

The strongest germline evidence currently comes from ICI-treated cohorts rather than studies designed to isolate a radiation interaction. Groha et al. identified an association between rs16906115 at *IL7* and all-grade immune-related adverse events (irAEs) in a GWAS of 1, 751 patients across 12 cancers, with a reported combined hazard ratio of 2.1 and replication in three independent studies. The locus colocalized with altered *IL7* splicing and was associated with maintained lymphocyte counts after ICI initiation (64). In a companion study of 214 patients with melanoma, Taylor et al. linked the same allele to severe irAE risk, higher *IL7* expression in B cells, and altered CD8-positive T-cell responses (65). The splicing and cell-specific expression observations support complementary regulatory hypotheses rather than a single established mechanism in irradiated tissue.

Further clinical support was reported in a 2025 retrospective melanoma cohort of 96 patients: rs16906115 minor-allele carriers had higher odds of all-grade irAEs (adjusted odds ratio 3.93, 95% confidence interval 1.13-13.64), although the wide interval indicates substantial uncertainty (66). *IL7* is therefore a credible candidate for prospective studies of radio-immunotherapy, but these estimates should not be applied to radiation pneumonitis or late fibrosis without endpoint-specific validation. In particular, an association with pooled irAEs does not identify which irradiated organ is vulnerable.

Organ-specific genetic evidence provides a second line of investigation. In 42 patients with high-PD-L1 non-small-cell lung cancer receiving pembrolizumab, pneumonitis occurred in 7 of 22 carriers of HLA-DPB1*02 and none of 20 noncarriers. The small number of events, multiple HLA comparisons, and absence of an independently replicated radiotherapy interaction make this finding exploratory (67). A hypothyroidism polygenic score was also associated with atezolizumab-related thyroid dysfunction in an analysis of 2, 616 genetically characterized patients across seven trials (68). Together, these studies support inherited variation in immune tolerance and antigen presentation, while showing why lung, thyroid, bowel, skin, and cardiac outcomes should not be represented by one undifferentiated genetic toxicity score.

A mechanistic model for combined treatment is that radiation determines the location and magnitude of tissue damage, whereas germline variation modifies the response to released antigens and inflammatory signals. Impaired DNA repair may prolong danger signaling; HLA variation may alter presentation of tissue-derived peptides; and *IL7*-related lymphocyte homeostasis may influence immune expansion after checkpoint blockade (13, 41, 47, 64, 65). Whether these processes interact synergistically, additively, or differently by organ and treatment sequence remains a testable hypothesis. Rare repair-gene defects and common immune-regulatory alleles should therefore be analyzed separately before their joint effects are modeled; neither pathway membership nor tumor radiosensitivity demonstrates inherited susceptibility to combined-treatment toxicity.

Phenotype adjudication is essential to testing this hypothesis. Radiation-associated pneumonitis more often has sharply demarcated, unilateral changes, whereas ICI-associated pneumonitis more often has bilateral, multifocal involvement; patients exposed to both treatments show overlapping patterns (69). Serial computed tomography (CT) registered to dose maps can support attribution, but imaging alone cannot prove etiology. Studies should distinguish field-concordant injury, diffuse inflammatory events, and uncertain overlap, while documenting infection, tumor progression, baseline interstitial abnormalities, pulmonary function, chemotherapy, and the timing of ICI exposure. This provides a practical connection between germline studies and imaging-based phenotyping (Section 4.5).

A variant associated with toxicity among patients receiving both treatments is a risk marker in that setting, not necessarily a modifier of the effect of adding radiotherapy or ICI. Establishing effect modification requires a treatment comparator, preferably within prospectively genotyped randomized trials, or carefully controlled cohorts with sufficiently comparable exposures. Analyses should prespecify genotype-by-treatment and genotype-by-organ-dose terms, report absolute risks on a defined time scale, and account for delayed ICI initiation and competing events. Serial lymphocyte and cytokine measurements may test mediation but should not be indiscriminately adjusted away as baseline confounders. Independent validation must retain the organ, toxicity grade, treatment sequence, and genetic model. The evidence reviewed here does not establish a germline test for withholding effective radio-immunotherapy or prescribing preventive immunosuppression (47, 63–68).

### 3.3 Oxidative stress, mitochondrial function, and redox defense

Radiation-induced reactive oxygen species damage DNA, proteins, lipids, mitochondria, and cell membranes. Enzymes and regulators including SOD1, SOD2, glutathione-dependent systems, GPX4, and the Keap1-Nrf2 pathway contribute to redox homeostasis (70–73). Variants that alter expression, localization, or activity of these components could plausibly modify the reserve available to normal tissues after irradiation. The direct clinical evidence for many such variants is limited, however. Studies of phototoxicity, environmental stress, bacteria, or tumor-directed ferroptosis can illustrate general redox principles but should not be treated as proof of inherited susceptibility to radiotherapy toxicity (70–72).

The most defensible interpretation is therefore mechanistic: reduced antioxidant capacity may increase the persistence of radiation-induced molecular injury, whereas effective redox responses may facilitate recovery. Establishing a radiogenomic effect requires more than measuring oxidative markers after irradiation. Investigators must demonstrate that the germline allele changes a relevant molecular or cellular phenotype and that the same allele is associated with an organ-specific clinical outcome (70–80).

Normal-tissue models show that radiation can increase lipid peroxidation products, deplete glutathione, impair mitochondria, and activate DNA damage signaling (73–76). Interventions that activate Nrf2 or reduce oxidative stress can attenuate injury in experimental skin, intestinal, hematologic, or hepatic models (73, 76, 78, 79). These experiments support redox defense as a modifiable component of tissue response. They do not establish that a particular SNP predicts clinical benefit from an antioxidant intervention. Translation requires genotype-stratified experiments, pharmacokinetic and safety assessment, and demonstration that protecting normal tissue does not also protect the tumor.

Oxidative stress and inflammation form a reciprocal amplification loop. Reactive oxygen species activate NF-kB and inflammasome-related signaling, while recruited neutrophils and macrophages generate additional oxidants (6, 76–79). Mitochondrial injury can also release immunostimulatory DNA and metabolites, connecting redox failure with innate sensing. This interaction may explain why early molecular damage progresses to prolonged symptoms or fibrosis after physical exposure has ended. Integrated studies that collect genotype, organ dose, longitudinal immune and oxidative biomarkers, and patient-reported outcomes are needed to determine whether redox-related variants alter immune amplification independently of exposure.

Mitochondria may integrate several toxicity pathways. Radiation can alter mitochondrial DNA, membrane potential, metabolism, and release of inflammatory signals. Variation in nuclear genes governing mitochondrial quality control could therefore influence redox reserve, senescence, and immune activation. This hypothesis is biologically coherent, but few loci have completed the full chain from human association to variant-specific mitochondrial phenotype and organ toxicity. It can remain a research priority rather than a basis for current treatment selection (70–80).

### 3.4 Apoptosis, autophagy, senescence, and tissue remodeling

Radiation changes cell-fate decisions in normal tissues as well as tumors. Apoptosis can remove heavily damaged cells and limit propagation of genomic instability, yet excessive apoptosis in rapidly renewing mucosa or intestinal epithelium can produce acute toxicity. Conversely, insufficient elimination may allow damaged cells to persist and release inflammatory mediators (81–83). TP53 and BCL-2 family signaling provide a plausible framework, but somatic TP53 mutations in cancer cells should not be used as direct evidence of germline normal-tissue susceptibility. Clinical radiogenomic claims require inherited variants, normal-tissue biology, and a defined toxicity endpoint.

Autophagy removes damaged proteins and organelles, recycles substrates, and supports homeostasis under cellular stress. Depending on dose, timing, and cell type, it may protect cells or contribute to cell death. Studies combining PARP inhibitors with photon or carbon-ion irradiation demonstrate that autophagy can modify tumor radiosensitivity (84). Their relevance to normal-tissue toxicity is indirect unless the same perturbation is examined in the exposed organ. Candidate variants in autophagy-related genes can therefore be evaluated in irradiated normal epithelial, endothelial, or stromal models rather than inferred from tumor-cell response alone.

Cellular senescence and the senescence-associated secretory phenotype are particularly important for late toxicity. Senescent epithelial, endothelial, stromal, and immune cells can secrete inflammatory cytokines, chemokines, growth factors, and matrix-remodeling enzymes that sustain leukocyte recruitment and fibroblast activation (13, 81–83). Immune surveillance may remove senescent cells and permit repair, whereas immune exhaustion, altered macrophage states, or persistent damage sensing may allow a chronic secretory program to develop. This framework links DNA repair, mitochondrial stress, innate sensing, adaptive immunity, and fibrosis, but variant-specific evidence remains limited.

Late fibrosis emerges from interactions among senescent cells, macrophages, lymphocytes, vascular injury, fibroblast activation, and extracellular matrix cross-linking (7, 42). Immune-cell and mediator profiles may be reparative in one phase and profibrotic in another, making time and tissue compartment essential experimental variables. A genetic variant may act at one step yet produce a clinical effect only above a dose threshold or in the presence of diabetes, smoking, surgery, or systemic therapy. Spatially resolved immune-stromal models are especially valuable because both radiation dose and cellular composition vary within an organ (35, 42, 57, 81–84).

### 3.5 From association locus to causal mechanism

Moving from a locus to a mechanism requires disciplined variant prioritization. The lead SNP from a GWAS is a statistical marker, not necessarily the functional allele. Fine mapping can define a credible set, after which regulatory annotation, conservation, transcription-factor binding, chromatin state, and three-dimensional contact data can identify plausible targets. Because many toxicity loci are noncoding, assuming that the nearest gene is causal can misdirect experiments. Multiple candidate genes may need to be tested, especially when the locus affects long-range enhancer activity (19, 22–24, 26, 27, 35).

Expression quantitative trait locus analysis can connect genotype to RNA abundance, but tissue and context matter. Whole-blood eQTLs may reflect systemic immune regulation yet miss response eQTLs in irradiated epithelium, endothelium, fibroblasts, resident macrophages, or organ-specific parenchyma. A variant may have no detectable baseline effect and become active only after DNA damage, cytokine exposure, or immune-cell contact. Genotype-stratified time courses and single-cell or spatial analyses can distinguish a cell-intrinsic regulatory effect from a change in tissue cellular composition (19, 22–24, 26, 27, 35, 85).

Chromatin architecture adds a physical link between a noncoding locus and its possible target genes. This is already used for annotation in toxicity radiogenomics: the oral-mucositis GWAS by Yang et al. incorporated Hi-C chromatin-interaction maps alongside positional and eQTL mapping within FUMA (19). The available maps covered 21 tissues or cell types and had approximately 40-kb resolution, so an interacting region could contain several genes. Enhancer and promoter annotations helped prioritize candidates, but these reference contacts were not measurements of allele-dependent looping in irradiated oral mucosa. The study therefore illustrates a current application to locus-to-gene prioritization and the limits imposed by tissue mismatch and contact-map resolution (19).

For fine-mapped toxicity loci, promoter-capture Hi-C or enhancer-associated contact assays can nominate distal promoters that would be missed by assigning each SNP to its nearest gene. Functional prioritization is strengthened when a credible-set variant overlaps an accessible, H3K27ac-marked enhancer that contacts a gene expressed in the affected normal-tissue cell type. The activity-by-contact framework formalizes this integration by combining enhancer activity with contact frequency and has been benchmarked against CRISPR perturbations (86). Applying such models to radiogenomic loci requires appropriate epithelial, endothelial, stromal, or immune reference maps. A detectable chromatin loop supports physical proximity; enhancer perturbation and target-gene measurements are needed to establish its regulatory consequence.

Allele-specific chromatin accessibility offers a complementary test of cis-regulatory activity. In heterozygous samples, ATAC-seq reads overlapping informative variants can reveal unequal accessibility of the two haplotypes within the same cellular environment, reducing some between-individual confounding. Reliable inference requires adequate allele-specific coverage, phasing where needed, and control of reference-mapping bias, for example with WASP; otherwise technical allelic imbalance can resemble a regulatory effect (87). Comparisons should be made within defined cell populations because bulk tissue mixtures can conceal or distort cell-specific effects. Accessibility quantitative trait loci, eQTLs, and toxicity associations should then be evaluated for colocalization, allowing for multiple signals at a locus. Allelic imbalance alone identifies a regulatory haplotype and does not resolve the causal nucleotide among linked variants.

Radiation exposure must be incorporated into this regulatory analysis. In stimulated macrophages, Alasoo et al. showed that shared genetic effects on chromatin accessibility and expression can reflect enhancer priming before an immune response (88). Although that study did not examine irradiation, it supports testing whether radiation or subsequent inflammatory signaling unmasks a previously silent regulatory effect. Paired pre-irradiation and post-irradiation ATAC-seq, contact mapping, and transcriptional profiling in relevant normal-tissue models could distinguish constitutive from response-dependent effects. Allele editing or enhancer CRISPR interference, followed by target-gene rescue and measurement of regeneration, barrier integrity, or matrix deposition, would test the proposed variant-enhancer-gene-toxicity chain. In the radiogenomic studies discussed here, reference-map annotation is more established than direct demonstration of radiation-dependent, allele-specific chromatin remodeling; clinical translation requires the latter mechanistic work together with cohort validation (19, 86, 88).

Transcription-factor occupancy can further connect candidate regulatory variants to inflammatory signaling. Aptamer-guided chromatin cleavage and sequencing (AptaChC-seq) maps transcription-factor binding through aptamer-directed Tn5 tagmentation and has been demonstrated for FOXM1, RELA, and RBPJ (89). For a fine-mapped variant overlapping a RELA-responsive element, paired occupancy and expression measurements before and after irradiation could test whether altered NF-kB binding accompanies the allelic response. This prospective application requires validation of aptamer specificity and allelic mapping in the selected normal-tissue model; the assay itself does not establish a variant-to-radiotoxicity relationship.

Molecular mediation can be tested explicitly. If a variant is associated with fibrosis and with expression of a matrix-remodeling gene, mediation analysis can estimate whether the molecular trait accounts for part of the clinical association. The assumptions are demanding: exposure, mediator, outcome, and confounders must be measured appropriately, and reverse causation must be considered when the mediator is collected after toxicity begins. Experimental perturbation remains necessary to support causal interpretation (19, 22–24, 26, 27, 35).

Cell composition can confound tissue-level molecular measurements. An apparent cytokine eQTL may reflect genotype-associated immune-cell infiltration rather than altered transcription within resident cells. Single-cell and spatial methods can separate epithelial, endothelial, myeloid, lymphoid, and stromal compartments and identify where a variant-dependent state emerges after irradiation. This resolution is particularly valuable for late toxicity, in which small populations of senescent cells, activated macrophages, or profibrotic fibroblasts may organize a much larger remodeling response (7, 13, 42, 85).

Targeted regional profiling offers a complementary option when prespecified transcripts and anatomical compartments are the main interest. Gel-anchored, ligation-assisted, scalable spatial sequencing (GLASS-seq) couples *in situ* probe ligation with hydrogel anchoring to quantify selected transcripts at approximately 100-µm lateral resolution (90). Applied to dose-registered normal-tissue specimens, it could help compare inflammatory and stromal programs across irradiated regions. Regional readouts require cell-composition controls and do not by themselves establish allele-specific regulation; application to radiation toxicity remains to be validated.

Temporal ordering provides another test of mechanism. DNA damage signaling occurs within minutes to hours, acute inflammation develops over hours to weeks, and fibrosis may emerge months or years later. A proposed pathway can explain how an early variant-dependent event persists, is remembered epigenetically, or triggers a self-sustaining tissue program. Cross-sectional measurements obtained only after established fibrosis cannot determine whether the signal caused injury or resulted from it. Longitudinal biospecimens and intermediate phenotypes can bridge these timescales (19, 22–24, 26, 27, 35).

Organ specificity can be treated as biological information rather than nuisance heterogeneity. A DNA repair variant might influence several acute endpoints, whereas a variant affecting a tissue-specific enhancer may act only in bladder, breast, lung, or salivary gland. Shared and organ-specific analyses are complementary. Cross-cancer meta-analysis can identify common host responses (27), while stratified models can preserve effects that would be diluted in a composite score. Formal genetic-correlation analysis may help determine which endpoints share architecture.

Mechanistic evidence can be graded. Replicated clinical association with variant-specific functional evidence in relevant normal tissue represents stronger support than a pathway paper without a human variant. An animal knockout establishes gene function but may provide only moderate support for a common regulatory SNP. Tumor-cell pharmacology or unrelated oxidative injury models provide indirect plausibility. Explicit grading prevents a long reference list from creating the impression that all links are equally established (19, 22–24, 26, 27, 35).

Mechanism also guides intervention. If a risk allele reduces repair capacity, the safest response may involve dose optimization rather than further inhibition of that pathway. If a locus promotes persistent inflammation or fibrosis, a targeted preventive agent could be tested in genotype-enriched cohorts. These strategies remain hypothetical until the variant identifies differential benefit or harm and the intervention preserves tumor response. A mechanistic narrative is clinically useful only when it leads to a testable and safe decision (19, 22–24, 26, 27, 35).

### 3.6 Acute, late, shared, and organ-specific mechanisms

The mechanistic sequence differs between tissues with rapid turnover and tissues dominated by slow remodeling. Oral and intestinal epithelia can develop symptoms when progenitor-cell loss, barrier failure, microbial signals, and innate immune recruitment outpace regeneration. Lung, heart, bladder, and connective tissues may show delayed dysfunction after endothelial injury, persistent macrophage and lymphocyte signaling, senescence, and matrix accumulation (4, 6, 7, 41, 42). Genetic studies should therefore match the proposed immune mechanism to organ anatomy and cellular kinetics rather than applying one generic inflammatory-radiosensitivity model.

Endothelial injury contributes to late toxicity through mechanisms that are partly distinct from loss of epithelial regenerative capacity. Barrier disruption, leukocyte adhesion, a procoagulant state, and progressive microvascular dysfunction can sustain inflammation and impair perfusion (59, 91). In the bowel, vascular sclerosis and ischemic injury contribute to chronic dysfunction, while fragile telangiectatic vessels are associated with bleeding; these manifestations should not be treated as interchangeable with fibrotic narrowing (92). A vascular-homeostasis variant would therefore be expected to influence endpoints such as permeability, perfusion, or hemorrhage, although this remains a hypothesis until tested for the individual locus. Endothelial models under relevant flow conditions can help separate a direct vascular effect from secondary inflammation or fibrosis.

The importance of this mechanical context is illustrated by a three-layer vascularized chip in which chamber geometry and interstitial flow influenced microvascular development, with the resulting networks supporting perfusion (93). Such engineering evidence supports controlling flow and geometry when testing candidate vascular-risk alleles. Adaptation to fractionated irradiation could help distinguish barrier failure and impaired vascular recovery from secondary stromal remodeling, but the chip study itself did not establish a germline effect on radiation injury.

The stem-cell reserve hypothesis provides a complementary explanation for organ-specific genetic effects. It proposes that the abundance of surviving, functionally competent stem and progenitor cells relative to regenerative demand determines how strongly a modest allelic effect is expressed at the organ level. A variant that increases apoptosis or senescence may have a larger clinical effect when irradiation depletes a small or spatially concentrated reserve, whereas a larger surviving pool or recruitment of alternative progenitors may buffer the same molecular defect. Reserve nevertheless depends on cell state, lineage plasticity, niche integrity, and the spatial dose distribution as well as cell number; a large but rapidly cycling pool is not necessarily radioresistant. Thus, inter-organ differences in reserve offer a testable explanation for effect heterogeneity, rather than evidence that any particular germline association is mediated by stem-cell abundance (94, 95).

Experimental and clinical observations support elements of this framework. In mice, depletion of Lgr5-positive intestinal stem cells severely impaired regeneration after irradiation despite compensation during uninjured homeostasis (94). In salivary glands, van Luijk et al. identified a stem-cell-rich ductal region and linked regional irradiation to subsequent functional loss (95). However, a later randomized trial involving 102 patients did not demonstrate a significant improvement in its primary parotid-function endpoint with stem-cell-region sparing (96). These findings support investigating the location and functional capacity of regenerative reserves while cautioning against assuming that regional sparing, or a germline effect on reserve, is already clinically established. Genotype-stratified organoid recovery, lineage tracing, and long-term clonogenic assays should therefore complement acute cell-death measurements, with parallel assessment of niche injury and organ function.

Stromal remodeling produces a different, although interacting, set of late phenotypes. Persistent fibroblast activation, myofibroblast differentiation, and extracellular matrix deposition can cause tissue induration, reduced compliance, and bowel stricture (42, 92). Collagen cross-linking further alters stiffness and mechanotransduction, helping to sustain remodeling after the initiating injury. The *KDM3B*-*LOX* example connects a radiogenomic signal to this matrix-regulatory process (35). Vascular rarefaction can worsen hypoxia and regenerative-niche dysfunction, while accumulating matrix can further compromise perfusion; endothelial, progenitor, and stromal mechanisms therefore converge without being biologically interchangeable (91, 92). Mechanistic validation should match the clinical phenotype: vascular permeability or perfusion for vascular injury, durable progenitor output for regenerative failure, and collagen organization or tissue mechanics for fibrosis. Combining these outcomes into a single late-toxicity grade could obscure distinct variant effects.

Systemic host factors may connect apparently distant endpoints. Baseline inflammatory state, metabolic disease, aging, immune composition, mitochondrial function, and prior immune-modulating treatment affect multiple organs. Cross-cancer analyses can detect shared susceptibility, but systemic effects should be distinguished from correlated treatment exposures. Multivariate models of several toxicities, paired with longitudinal immune phenotyping, may identify a common host-response component while preserving organ-specific residual effects (4, 6, 27, 56, 59, 62, 97).

Mechanistic uncertainty can shape wording. Terms such as mediates, determines, and causes can be reserved for evidence that supports intervention or causal inference. Association, colocalization, pathway enrichment, and experimental perturbation answer different questions. Using precise verbs makes the review more informative: a locus can be associated with an endpoint, a variant can alter expression, a pathway can be activated by radiation, and a perturbation can modify injury without any single study proving the complete clinical chain (27, 35, 56, 59, 62, 97).

## 4 Polygenic risk scores and integrated toxicity prediction

From an immunological perspective, prediction should distinguish stable inherited predisposition from dynamic immune state. Germline scores may estimate baseline susceptibility, whereas cytokines, immune-cell states, and tissue imaging collected after treatment may reflect exposure, mediation, or early injury. Combining these variables without respecting time order can create leakage and obscure whether a model predicts risk or detects toxicity already underway.

### 4.1 Principles, construction, and limitations of polygenic risk scores

A polygenic risk score aggregates alleles across the genome, usually weighting each allele by an effect estimate derived from GWAS summary statistics (98, 99). The approach is attractive for radiotherapy toxicity because most common variants are expected to have small effects and multiple biological pathways contribute to injury. A toxicity-specific score must be trained on the same or a closely related endpoint; a score for developing cancer does not automatically measure radiosensitivity. Indeed, cancer-susceptibility polygenic scores were not associated with radiotherapy toxicity in an analysis of breast, prostate, and lung cancer cohorts (9). This negative result is important because it defines the construct that a clinically useful score must measure.

Simple scores use clumping and thresholding or weighted allele counts, whereas newer methods model linkage disequilibrium and prior distributions across many variants (98–103). LDpred, PRS-CS, SBayesRC, and related approaches can improve estimation when discovery samples are large and ancestrally matched. User-friendly repositories and imputation-server workflows reduce computational barriers (102, 103). Methodological sophistication cannot compensate for poor phenotype definition, small discovery cohorts, or unmodeled treatment exposure. A score trained on heterogeneous acute toxicity may not predict a specific late organ complication, even if both are labeled radiotherapy toxicity.

Ancestry transferability is a major concern because allele frequencies, linkage disequilibrium, environmental exposures, and clinical practice differ across populations (8, 101, 104, 105). Scores developed primarily in European-ancestry cohorts may lose discrimination or become miscalibrated in other groups. Multi-ancestry methods such as PRS-CSx and ancestry-aware standardization may improve portability, but local validation remains necessary (101, 105). Low-pass sequencing and imputation may reduce assay cost (103, 106); nevertheless, analytical validity, genotype quality, calibration, and data governance must be addressed before clinical implementation. A standardized z score is not itself an absolute probability of toxicity.

A score intended for clinical use needs a clearly specified target population and outcome. Developers can report the training ancestry, cancer site, treatment era, endpoint, follow-up, number of events, variant-selection method, and reference panel. Validation can assess both discrimination and calibration in patients receiving contemporary radiotherapy. If the baseline incidence differs from the development cohort, recalibration may be necessary even when the relative ranking of risk is preserved (9, 22, 27, 98–107).

Clinical utility also depends on the consequences of error. A false-high classification could lead to unnecessary dose compromise, anxiety, or avoidance of curative treatment; a false-low classification could reduce monitoring or supportive care. These asymmetric harms can guide threshold selection and shared decision-making. Until prospective studies define safe actions, toxicity PRS can be considered investigational and should not supersede accepted organ-at-risk constraints (9, 22, 27, 98–107).

### 4.2 Development and validation of clinical-genomic toxicity models

A clinically useful toxicity model can combine inherited susceptibility with variables that determine tissue exposure, immune context, and baseline reserve. These include organ dose-volume metrics, fractionation, treatment technique, concurrent systemic therapy, prior surgery, age, comorbidity, smoking, autoimmune disease, infection history, organ function, and patient-reported baseline symptoms (11, 21, 39, 40, 108). Longitudinal cytokine or immune-cell measurements may improve dynamic prediction, but post-treatment biomarkers must be distinguished from baseline predictors and mediators. Genomic information is valuable only if it improves prediction beyond established clinical and dosimetric factors and remains calibrated across treatment contexts.

Model development may use logistic regression, time-to-event analysis, competing-risk models, penalized regression, gradient boosting, or other machine-learning methods (108). The algorithm is less important than design quality. Cohorts can define the prediction time point, candidate predictors, missing-data strategy, and validation approach before model fitting. Repeated cross-validation can reduce optimism during development but does not replace external validation in a geographically or temporally distinct cohort. When sample size is limited, complex models can overfit noise and produce apparently high discrimination that disappears in new patients.

Evaluation can include calibration-in-the-large, calibration slope, discrimination, confidence intervals, and clinically relevant risk thresholds. Net reclassification or a small increase in area under the curve is insufficient if the model does not change management. Decision-curve analysis can estimate whether using the model produces net benefit across plausible thresholds, but a prospective trial is needed to show that model-guided care reduces toxicity without compromising tumor control. Potential actions include adaptive planning, modified organ-at-risk constraints, intensified supportive care, avoidance of high-risk drug combinations, or closer surveillance. Each action requires its own evidence and safety limits (**Figure 4**) (9, 11, 12, 21, 26, 27, 98–109).

**Figure 4:**
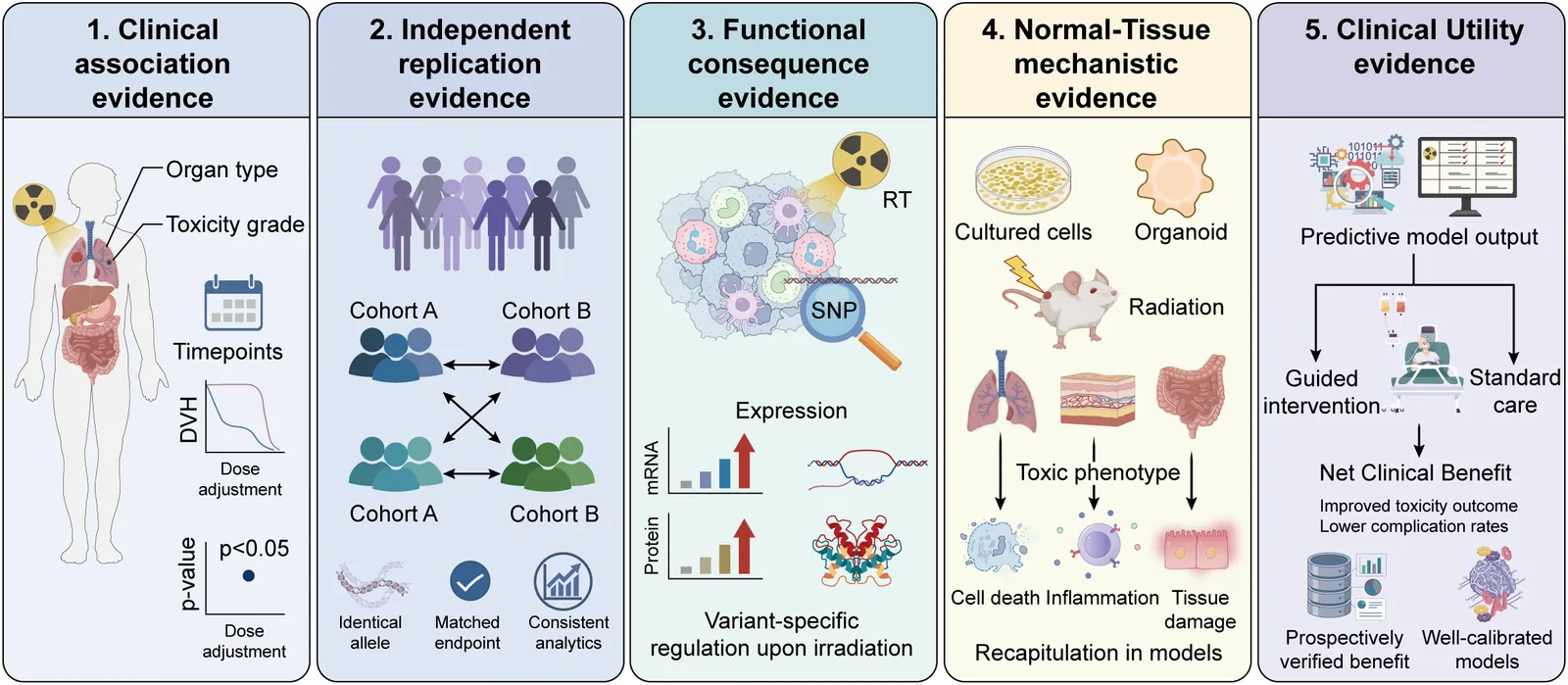
Development and evaluation of an integrated clinical-genomic model for radiotherapy toxicity.

Transparent reporting is essential because multimodal models can hide sources of leakage and optimism. Feature selection, normalization, imputation, and hyperparameter tuning must be performed within the training data rather than before data splitting. Patients, not images or repeated visits, can define the unit of resampling. External validation can report performance by cancer site, ancestry, sex where relevant, treatment technique, and toxicity prevalence. Model cards or equivalent documentation can make intended use and limitations explicit (9, 11, 12, 21, 26, 27, 98–109).

Implementation requires integration with treatment planning and clinical workflows. A risk estimate can be available at the time a decision is made, use data that can be obtained reliably, and present uncertainty in a form clinicians and patients can understand. Genomic testing also raises questions of consent, privacy, incidental findings, data retention, and access. These operational requirements are part of clinical validity rather than secondary administrative details (9, 11, 12, 21, 26, 27, 98–109).

### 4.3 Clinical utility, ethics, and implementation

Clinical validity and clinical utility are distinct. A model is clinically valid when it predicts the intended outcome accurately in the target population. It is clinically useful when acting on its prediction improves outcomes compared with usual care. A perfectly calibrated risk estimate may have little utility if no safe intervention exists, while a moderately discriminative model may be useful if it identifies patients who benefit from low-risk preventive care. Development reports can state the intended action rather than presenting prediction as an end in itself (8, 9, 26, 27, 101, 105, 108).

Treatment planning is a constrained optimization problem balancing tumor control and normal-tissue risk. Genomic information could, in principle, modify the estimated normal-tissue complication probability curve or the uncertainty around it. Before this is attempted clinically, models must show that genotype adds information beyond three-dimensional dose and established clinical factors. They must also define how much risk change is sufficient to alter a constraint. Unvalidated genomic adjustment of dose could reduce cure probability and is not justified by the current evidence (8, 9, 26, 27, 101, 105, 108).

Near-term applications may be less invasive than dose modification. A high-risk estimate could trigger dental, pulmonary, gastrointestinal, cardiac, skin, or rehabilitation assessment; closer monitoring of patient-reported outcomes and inflammatory symptoms; earlier diagnostic evaluation; avoidance of poorly supported immune-toxic combinations; or enrollment in prevention trials. A mechanistically defined immune-risk profile might also support genotype-enriched trials of anti-inflammatory, senolytic, or antifibrotic interventions, provided tumor response is protected. Even low-risk applications require evidence that alerts are accurate enough to avoid unnecessary visits, immunosuppression, cost, and anxiety (8, 9, 26, 27, 101, 105, 108).

Communication of genomic risk requires care. Relative risk, percentile rank, and absolute probability convey different information. Patients can understand that common variants modify probability rather than determine outcome, and that dose, comorbidity, and chance remain important. Uncertainty intervals and limits of ancestry transferability can be visible. Decision aids and genetic counseling may be appropriate when testing includes rare pathogenic variants or incidental findings beyond the toxicity question (8, 9, 26, 27, 101, 105, 108).

Genomic data are durable identifiers with implications beyond the treatment episode. Consent can address data storage, secondary research use, return of results, family implications, and potential reanalysis. Access controls and governance are particularly important for multi-institutional consortia. Equity requires more than diverse recruitment: testing costs, laboratory availability, digital infrastructure, and interpretation resources should not make precision toxicity prevention available only to selected centers or populations (8, 9, 26, 27, 101, 105, 108).

Prospective evaluation can use several designs. An observational validation cohort tests calibration and transportability under contemporary care. An impact study measures whether providing risk estimates changes clinician or patient decisions. A randomized utility trial compares model-guided management with standard care. Cluster or stepped-wedge designs may be useful when the intervention operates at the clinic level. Endpoints can include toxicity, tumor control, quality of life, resource use, and disparities (8, 9, 26, 27, 101, 105, 108).

Models will require maintenance as treatment and populations change. Recalibration can update the baseline risk, while model revision may be necessary when new techniques or systemic therapies alter predictor effects. Performance monitoring can detect drift and subgroup failure. A learning health-system approach can support updating, but updates must be versioned and validated before clinical use. Continuous learning should not become uncontrolled model change at the bedside (8, 9, 26, 27, 101, 105, 108).

### 4.4 Interpreting prediction performance

Discrimination describes ranking, not probability accuracy. The area under a receiver operating characteristic curve estimates how often a randomly selected patient with toxicity receives a higher score than one without toxicity. It does not show whether a predicted risk of 30% occurs in 30% of comparable patients. Calibration plots, intercepts, and slopes are therefore necessary for counseling and threshold-based decisions (9, 26, 27, 98, 101, 105, 107, 108).

Class imbalance can make accuracy misleading when severe toxicity is uncommon. A model that predicts no events may appear accurate while having no clinical value. Sensitivity, specificity, positive and negative predictive value, precision-recall curves, and event-based confidence intervals provide complementary information. Metrics can be selected according to the intended decision and reported at clinically plausible thresholds rather than only at a data-optimized cut (9, 26, 27, 98, 101, 105, 107, 108).

Incremental value can be evaluated against a strong baseline model. Comparing a genomic model with age alone can exaggerate benefit if dose-volume predictors are omitted. The reference model can include variables already available and accepted for the organ and treatment context. Genomic features can then be assessed through change in calibration, discrimination, explained variation, or net benefit. A statistically significant coefficient does not guarantee worthwhile incremental prediction (9, 26, 27, 98, 101, 105, 107, 108).

Missing data can introduce bias if genotyping success, toxicity follow-up, or dosimetric data are related to prognosis or treatment center. Complete-case analysis may discard informative patients and alter ancestry composition. Multiple imputation can be appropriate when assumptions are defensible, but genotype, outcome, and treatment variables require careful handling. Reports can describe missingness by cohort and compare included with excluded patients (9, 26, 27, 98, 101, 105, 107, 108).

Transportability can be assessed rather than assumed. A model may fail in a new cohort because of ancestry, endpoint definition, dose distribution, supportive care, or baseline incidence. Recalibration addresses some differences, while changed predictor effects require revision. Evaluating performance across several external cohorts reveals whether a model captures a stable host-response signal or local practice. Heterogeneity is a finding to explain, not merely an obstacle to obtaining a pooled performance number (9, 26, 27, 98, 101, 105, 107, 108).

Uncertainty in individual predictions can be acknowledged. Genomic effect estimates, baseline risk, and treatment exposure are all measured with error. Presenting a single precise percentage can imply confidence that the data do not support. Prediction intervals, risk categories with explicit uncertainty, and sensitivity to model assumptions may better support shared decisions. Uncertainty is particularly important when a proposed action could alter curative treatment (9, 26, 27, 98, 101, 105, 107, 108).

### 4.5 Integrating germline susceptibility with imaging-based radiogenomics

Integration is most informative when each modality retains its biological meaning. Germline genotypes describe stable host susceptibility; normal-organ radiomic features may capture tissue reserve or structural heterogeneity; and spatial dose features describe exposure. Tumor imaging-genomic signatures address a different component, namely tumor biology and expected treatment benefit (11, 12, 110). A joint framework could therefore model tumor control and normal-tissue complications as separate, clinically linked outcomes. Tumor mutation status, transcriptomic radiosensitivity, or an imaging surrogate for these features should not be relabeled as inherited toxicity risk. Paired normal DNA is needed when sequencing data require separation of somatic and germline findings.

There is preliminary evidence that genetic and spatial exposure information can be complementary. Liang et al. developed a model in 400 patients receiving thoracic radiotherapy, including 108 with grade ≥2 pneumonitis. Adding four dosiomic features to a 19-SNP interaction model increased the testing-set area under the receiver operating characteristic curve from 0.76 to 0.81 (111). This is a proof of concept for germline-dosiomic modeling, not external validation of a complete germline-radiomic predictor. Dosiomics quantifies the dose distribution, whereas radiomics characterizes image-derived tissue features. The prostate dose-surface study similarly links genotype with spatial exposure (21); neither result establishes that tumor imaging features can substitute for host genotyping.

A prospective integration study should obtain baseline normal-organ and tumor images, germline DNA, and the planned three-dimensional dose distribution before treatment. Organ segmentation and dose registration would permit evaluation of genotype-associated vulnerability within specific subregions. Subsequent imaging could characterize injury trajectories and provide intermediate phenotypes for genotype-to-tissue-response analyses. A proposed lung model, for example, could test whether an immune-regulatory allele is associated with greater parenchymal change at comparable local dose, followed by symptomatic pneumonitis. Such mediation requires temporal ordering and control of shared causes; a genotype-imaging correlation alone is insufficient. Post-treatment changes belong in prespecified landmark or dynamic models rather than a pretreatment risk score (69, 108, 110).

Model architecture should reflect cohort size and the decision being addressed. Early fusion combines selected, standardized genomic, imaging, and dose features in one penalized model; late fusion combines predictions from separately developed modality-specific models. The latter may facilitate evaluation when one modality is unavailable, but requires a prespecified missing-modality strategy. Flexible interaction models are attractive only when supported by enough independent patients and toxicity events (108). In either approach, comparing clinical-dosimetric, germline-augmented, imaging-augmented, and fully integrated models is necessary to establish whether the additional modality contributes useful information rather than redundant complexity (112).

Technical and population effects can otherwise masquerade as biological integration. Scanner vendor, reconstruction kernel, voxel size, contouring, and image registration alter radiomic measurements; Image Biomarker Standardization Initiative definitions and repeatability assessment provide an essential analytical foundation (113). Genotyping batch and ancestry may also correlate with imaging center, creating spurious genotype-feature associations. Harmonization, imputation, feature selection, and tuning must be learned within training data, with repeated images from each patient kept in the same partition. Statistical batch correction cannot rescue a design in which ancestry, center, and treatment are inseparable. External evaluation across institutions, scanners, ancestries, and contemporary systemic regimens is therefore required (112).

Federated reconstruction provides a methodological example of addressing imaging heterogeneity across institutions. The metadata-constrained FedM2CT framework combines task-specific CT reconstruction with mutual learning across differing acquisition geometries and sampling protocols (114). Its relevance to integrated radiogenomics lies in the upstream handling of heterogeneous images; reconstruction performance alone does not establish radiomic feature stability or genotype-to-toxicity prediction. Before adopting such methods, investigators should test preservation of normal-tissue texture, repeatability of extracted features, and transportability across scanners and dose levels, followed by independent validation of the complete toxicity model.

Clinical value should be judged by organ- and time-specific calibration, discrimination, uncertainty, and net benefit relative to an established clinical-dosimetric model, following transparent prediction-model reporting (112). Imaging-defined pneumonitis patterns may improve endpoint consistency during radio-immunotherapy, but a classifier of established injury is a diagnostic tool rather than evidence of pretreatment prediction (69). Near-term applications are prospective evaluation of surveillance and supportive-care strategies. Using an integrated model to alter tumor dose or select an ICI regimen would additionally require evidence that the resulting decision improves toxicity outcomes while preserving cancer control.

## 5 Functional validation and mechanistic translation

### 5.1 Isogenic cell systems and genome editing

Functional validation is the bridge between an association signal and a credible immune mechanism. Isogenic systems created with CRISPR/Cas9, base editing, prime editing, CRISPR interference, or CRISPR activation can isolate the effect of a candidate allele in a controlled background. Readouts should extend beyond clonogenic survival to damage sensing, cGAS-STING or inflammasome activation, cytokine and chemokine secretion, immune-cell recruitment signals, macrophage or lymphocyte responses, senescence, and extracellular matrix production. The cell type must match the toxicity phenotype, and immune-cell co-culture or conditioned-medium transfer may be required when the proposed effect is non-cell-autonomous (4–7, 13, 14, 35, 41, 42, 85).

An isogenic comparison reduces background genetic variation, but editing can introduce off-target effects or clonal artifacts. Experiments can include independently derived clones, rescue or reciprocal editing, blinded phenotyping, and verification that the edited allele changes the expected regulatory or protein function. Radiation dose and fractionation can approximate the biological context of the clinical endpoint (14, 35, 115–117).

The KDM3B locus provides a useful example of functional triangulation (35, 85). Human association and allele-specific experiments connected KDM3B single-nucleotide polymorphisms with circular RNA-mediated regulation, inflammatory responses, and radiotherapy toxicity; complementary work linked KDM3B regulation to LOX mRNA stability, collagen cross-linking, and post-radiation fibrosis (35, 85). This chain is more informative than pathway enrichment alone because it connects inherited variation to inflammatory signaling and tissue remodeling. Replication across cohorts and organ-relevant immune-stromal models remains necessary before clinical use.

The RNA-regulatory component of this mechanism also requires modification-specific measurements. Chemical-assisted assays can distinguish modified nucleic-acid bases that conventional sequence-complementarity assays cannot resolve, although conversion efficiency, sensitivity to low-abundance modifications, and loss of spatial information can limit interpretation (118). TadA8.20-assisted N^6^-methyladenosine RNA imaging at single-base resolution (TARS) quantified site-specific m^6^A modifications in MALAT1 and CCND1 transcripts in cancer-cell models (119). These approaches could support testing of RNA-modification mechanisms downstream of toxicity loci, including LOX-transcript regulation, but require adaptation to irradiated normal tissue. A treatment-associated methylation change is not itself evidence of inherited susceptibility; allele perturbation, temporal ordering, and functional rescue remain necessary.

Genome editing is therefore an experimental instrument for causal inference, not clinical validation by itself. Broader oncology applications of cell engineering and CRISPR in CAR-T, breast cancer, and glioblastoma demonstrate technical feasibility (115–117), but they do not provide direct evidence for inherited normal-tissue radiotoxicity. Keeping this distinction explicit prevents general advances in cancer biotechnology from being counted as support for a toxicity biomarker.

High-throughput functional screens offer a way to prioritize many candidate variants, but screening systems must be designed around normal-tissue phenotypes. Pooled perturbation screens can identify genes affecting survival or DNA repair after irradiation, while single-cell readouts can reveal cell-state-specific responses. Follow-up experiments can test the precise human allele and quantify effect size under clinically relevant dose conditions. A gene-level screen is not sufficient to validate a particular noncoding SNP (14, 35, 115–117).

### 5.2 Organoids, patient-derived models, and *in vivo* validation

Normal-tissue toxicity is an organ-level process involving epithelial, endothelial, stromal, vascular, immune, and neural compartments. Organoids and patient-derived normal-tissue cultures can preserve architecture, but many organoids lack resident or recruited immune populations. Immune-competent co-culture, vascularized organoids, air-liquid-interface systems, or microphysiological platforms can test genotype-dependent communication among parenchymal cells, macrophages, lymphocytes, endothelium, and fibroblasts. These models require standardized differentiation, cellular composition, radiation dosimetry, sampling time, and reproducibility across laboratories (4–7, 13, 14, 35, 41, 42, 51, 73, 76, 85).

Vascularized organ-on-chip designs incorporating microchannels, membranes, or hydrogels provide practical strategies for combining tissue architecture with controlled transport conditions (120). In radiogenomic experiments, such designs could be adapted to compare allele-edited endothelial and parenchymal compartments while measuring permeability, regenerative recovery, and matrix deposition. Their value would depend on reproducing organ-relevant cell states, flow, and radiation exposure; engineering feasibility alone does not validate a model of clinical toxicity.

Genetically engineered and conditional animal models can test whether a candidate pathway has cell-autonomous or immune-microenvironment-mediated effects. Tissue-specific deletion can separate epithelial from endothelial or fibroblast contributions; bone marrow chimeras and adoptive transfer can distinguish hematopoietic from tissue-resident effects; and immune-cell depletion can identify phase-specific roles. Models should reproduce the clinically relevant organ, fractionation, latency, and endpoint. A complete knockout or systemic immune depletion may establish pathway importance but can greatly overstate the effect of a common human regulatory allele (4, 14, 35, 41, 42, 51, 73, 76).

The strongest validation pipeline triangulates four layers: replicated clinical association, adjustment for organ dose and treatment context, variant-specific molecular perturbation, and organ-relevant validation (**Figure 5**). Concordance across layers increases confidence that the locus is not merely a statistical passenger. Discordant results are also informative: they may indicate that the index SNP tags another causal variant, that the relevant cell type was not modeled, or that the clinical association was unstable. Negative functional results can be reported rather than selectively omitted (14, 35, 51, 73, 76).

**Figure 5:**
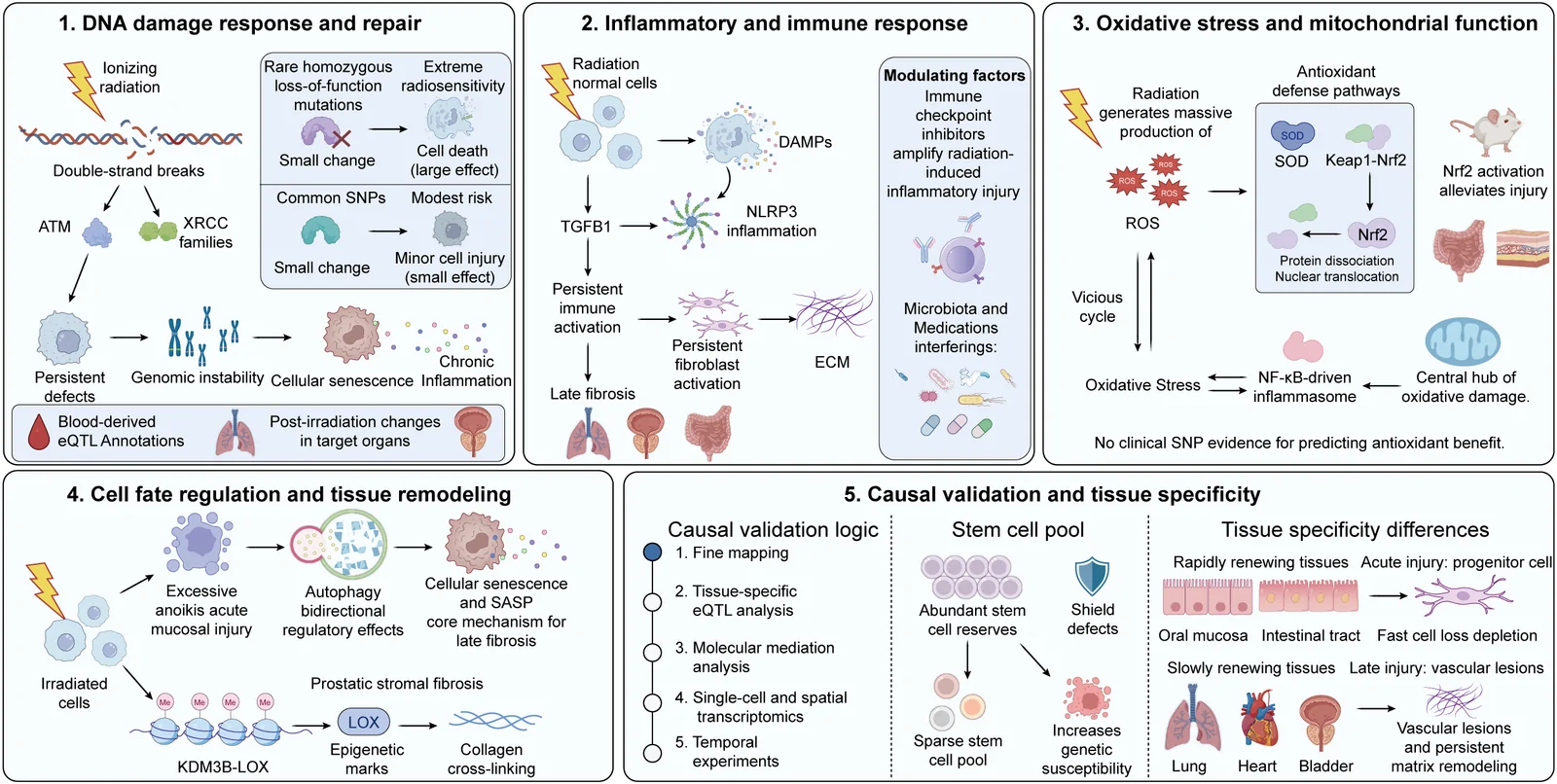
Functional validation ladder from variant prioritization to prospective clinical evaluation.

Translation to radioprotective interventions introduces an additional constraint: protection of normal tissue must not reduce tumor control. Candidate antioxidants, anti-inflammatory agents, senolytics, or antifibrotic therapies can be evaluated in paired tumor and normal-tissue systems. A genotype that identifies a vulnerable pathway may help enrich prevention trials, but genotype-guided therapy requires pharmacodynamic evidence, safety monitoring, and a prespecified interaction between treatment effect and genetic risk (14, 35, 51, 73, 76). For radio-immunotherapy candidates, a factorial design comparing each genotype under irradiation alone, checkpoint blockade alone, their combination, and matched controls could distinguish baseline susceptibility from treatment interaction. Immune-competent normal-tissue systems and paired tumor models are required to assess tissue-directed immune injury together with preservation of antitumor activity (40, 65).

Biobanks linked to radiotherapy plans, longitudinal toxicity assessments, and biospecimens can support this validation pipeline. Consent and governance can permit reproducible analysis while protecting genomic privacy. Data standards can preserve raw or derived dose information, treatment dates, fractionation, toxicity timing, and ancestry principal components. Without these details, future investigators may be unable to distinguish a biological nonreplication from an incompatible cohort (14, 35, 51, 73, 76).

### 5.3 Design principles for functional studies

Experimental design can begin with a causal model that states the proposed allele, target gene, cell type, molecular consequence, radiation interaction, and clinical endpoint. This prevents a broad assay panel from generating an attractive but *post hoc* story. The selected radiation schedule can represent the biological question: a single high dose may be useful for DNA damage kinetics, whereas fractionated exposure may better model senescence, recovery, and fibrosis. Unirradiated genotype effects and genotype-by-radiation interactions can both be estimated (14, 35).

Replication is as important in functional work as in association studies. Independent clones, donors, experimental days, and laboratories address different sources of variability. Sample size calculations can use biological replicates rather than counting technical wells as independent observations. Blinded outcome assessment, predefined exclusion rules, and reporting of all tested endpoints reduce bias. Rescue experiments can demonstrate that restoring the target gene or pathway reverses the variant-associated phenotype (14, 35).

Noncoding variants often require allele-specific assays. Reporter constructs can test enhancer activity but remove the sequence from its chromatin context. Editing the endogenous locus, chromatin accessibility assays, transcription-factor binding studies, and chromosome-conformation methods provide complementary evidence. Concordance across assays is more persuasive than a large effect in a single artificial system. The relevant allele orientation and haplotype can be verified before experiments are interpreted (14, 35).

Complex models can be introduced when they answer a defined mechanistic limitation. Co-culture can test epithelial-immune, endothelial-myeloid, or macrophage-fibroblast communication; organoids can model architecture and regeneration; microphysiological systems can incorporate perfusion and leukocyte trafficking; and animals can evaluate systemic and late effects. Sampling should be time resolved because innate sensing, lymphocyte recruitment, resolution, senescence, and fibrosis occur on different scales. No model reproduces the entire clinical phenotype, so triangulation across allele-aware systems is more realistic than seeking one definitive platform (4–7, 13, 14, 35, 41, 42, 85).

Extreme-phenotype sampling can increase efficiency. Patients with unexpectedly severe toxicity despite acceptable exposure, or unusually low toxicity despite high exposure, may be informative for rare-variant sequencing and patient-derived models (14). Selection must account for dose, comorbidity, medication, and follow-up to avoid labeling exposure differences as biology. Findings from extremes can then be evaluated in unselected cohorts before population-level risk is estimated.

Functional results can feed back into human analysis. A validated target gene can motivate burden testing of rare variants, pathway-based polygenic scores, or prespecified interaction analysis. Conversely, lack of a functional effect may redirect fine mapping or identify an incorrect tissue model. This iterative loop between cohort and laboratory is more reliable than a linear pipeline in which every association is forced into the first plausible pathway (14, 35).

## 6 Challenges and future directions

The principal barrier to translation is not a shortage of plausible immune pathways but insufficient convergence of evidence. Many reported variants originate from small retrospective cohorts and have not been tested independently, while broad evidence that radiation activates inflammation is sometimes used to overstate the relevance of a particular allele. Consortia can adopt prospective analysis plans, shared organ- and time-specific phenotypes, dose harmonization, and mechanisms for publishing null association and functional results. Replication should be evaluated at the variant, immune-response phenotype, locus, pathway, and prediction-model levels (4–9, 13, 21, 22, 26, 27, 35, 41, 42, 85, 101, 105, 108).

Treatment evolution creates another challenge. Intensity-modulated radiotherapy, particle therapy, adaptive planning, altered fractionation, and immune-modulating systemic combinations change both tissue exposure and inflammatory context. A marker discovered in an older treatment era may retain biological meaning but lose predictive value when exposure is reduced, while a variant affecting immune regulation may become more relevant during checkpoint blockade. Temporal validation, explicit treatment-interaction analysis, and model updating are therefore necessary (8, 9, 21, 22, 26, 27, 35, 39, 40, 101, 105, 108).

Phenotypic measurement can combine clinician grading, objective organ function, and patient-reported outcomes. Clinician grades may underrepresent persistent symptoms, while patient reports can be influenced by baseline health and expectations. Rather than choosing one source as the universal standard, investigators can define complementary endpoints and analyze their genetic correlation. Longitudinal models may identify trajectories of onset, persistence, and recovery that are more biologically informative than maximum grade (8, 9, 21, 22, 26, 27, 35, 101, 105, 108).

Multi-omics integration may improve mechanism discovery, but it also increases dimensionality and risk of overfitting. DNA methylation, transcriptomics, proteomics, metabolomics, microbiome data, radiomics, circulating cytokines, and single-cell immune states can be informative when collected at appropriate time points (11, 12, 108, 109, 121). Germline variation is stable, whereas most immune and omics signals are dynamic consequences, mediators, or modifiers of treatment. Causal diagrams, repeated sampling, cell-composition adjustment, and mediation analysis can help distinguish baseline susceptibility from early injury and downstream biomarkers.

Immune phenotyping should be designed around mechanism rather than assay availability. Blood measurements may capture systemic responses but can miss organ-resident macrophages, tissue-resident lymphocytes, endothelial activation, and spatial immune-stromal niches. Paired blood and tissue sampling, when clinically feasible, plus single-cell or spatial profiling can identify response quantitative trait loci and cell-state transitions. These resource-intensive studies should prioritize replicated loci and prespecified time points so that descriptive immune atlases can be connected to inherited susceptibility and clinical endpoints.

Small extracellular vesicles provide another candidate source of longitudinal molecular readouts. A sharp-edge acoustofluidic platform demonstrated capture and fluorescence detection of epidermal growth factor receptor (EGFR)-positive vesicles from cultured-cell preparations using 50-µL samples (122). This offers a technical basis for evaluating low-volume serial assays, but the reported EGFR assay does not establish organ specificity or predict radiation injury. Prospective radiogenomic studies would need validated tissue-of-origin markers, standardized preanalytics, paired baseline and post-treatment sampling, and joint analysis with genotype and dosimetry before interpreting vesicle changes as mediators or early toxicity signals.

Prospective clinical utility studies are the decisive next step. Initial trials can test genomic- and immune-informed surveillance, supportive care, or prevention while maintaining accepted tumor-dose requirements. Genotype-enriched studies of anti-inflammatory, senolytic, or antifibrotic approaches should include pharmacodynamic immune readouts and confirm that normal-tissue protection does not impair tumor control. Outcomes can include toxicity, infection, immune-related adverse events, tumor control, quality of life, anxiety, resource use, and disparities. Dose de-escalation or target-volume change requires stronger evidence than is currently available (8, 9, 21, 22, 26, 27, 35, 42, 101, 105, 108).

This review is a narrative synthesis rather than a systematic review. It does not apply formal screening, risk-of-bias assessment, or pooled effect estimation. The included evidence spans clinical association studies, methodological papers, functional experiments, and broader radiobiological literature, so inferential strength differs across sections. Several references illustrate general mechanisms or modeling principles rather than direct variant-to-toxicity relationships. These limitations reinforce the need to label evidence as replicated association, functional support, indirect plausibility, or translational hypothesis (8, 9, 21, 22, 26, 27, 35, 101, 105, 108).

Consensus reporting standards would improve comparability. Every study can identify the toxicity scale and version, baseline assessment, follow-up window, organ definition, treatment technique, fractionation, dose-volume covariates, ancestry method, genetic model, multiple-testing threshold, and replication status. Prediction reports can additionally provide the complete model, calibration, external validation, and intended use. A concise evidence table can make these elements visible and prevent a nominally positive association from being interpreted without its clinical context (8, 9, 21, 22, 26, 27, 35, 101, 105, 108).

Sequencing should complement, rather than simply extend, common-variant scoring. Rare-variant masks and clinical classifications require gene- and inheritance-specific interpretation, while estimates from extreme-phenotype cohorts need validation in broader radiotherapy populations. Parallel collection of normal-organ images, dosimetry, and systemic-treatment timing would allow these distinct genetic architectures to be evaluated in the same prospective framework without assuming that a rare allele has a larger or more actionable effect (43, 44, 47, 48).

Sex and sex-specific anatomy may influence both exposure and biology. Some endpoints occur only in particular treatment populations, while hormonal state, body composition, and immune response can modify toxicity. Analyses can report sex and evaluate relevant interactions when power permits. This consideration should not be reduced to statistical adjustment: the biological and anatomical pathway linking genotype, treatment, and endpoint can be specified (8, 9, 21, 22, 26, 27, 35, 101, 105, 108).

Comorbidity can alter tissue reserve and competing risk. Diabetes, vascular disease, autoimmune conditions, chronic lung disease, renal impairment, and prior inflammatory injury may influence both treatment selection and toxicity. Genetic effects estimated in highly selected trial participants may not transport to routine practice with greater multimorbidity. Pragmatic cohorts and linkage to longitudinal clinical data can complement tightly controlled discovery studies (8, 9, 21, 22, 26, 27, 35, 101, 105, 108).

Causal-inference methods may help prioritize hypotheses but cannot replace design. Mendelian randomization, mediation analysis, colocalization, and genetic correlation each rely on specific assumptions. Horizontal pleiotropy, weak instruments, selection after cancer diagnosis, and treatment-dependent exposure can violate those assumptions. Results can be triangulated with functional and clinical evidence rather than presented as automatic proof of a therapeutic target (8, 9, 21, 22, 26, 27, 35, 101, 105, 108).

Data from biobanks require attention to selection. Patients who receive radiotherapy differ from all patients with cancer, and those with complete genotype, dosimetry, and long-term toxicity data may be further selected. Conditioning on treatment or survival can create collider bias. Explicit cohort diagrams, comparison with source populations, and sensitivity analyses help readers understand which population the estimated association represents (8, 9, 21, 22, 26, 27, 35, 101, 105, 108).

The field can distinguish biomarker qualification from exploratory research. An exploratory locus can guide experiments; an analytically validated assay reliably measures the genotype; a clinically validated biomarker predicts the specified endpoint; and a clinically useful test improves decisions. These stages require progressively stronger evidence. Labeling all associated SNPs as biomarkers compresses this pathway and overstates readiness (8, 9, 21, 22, 26, 27, 35, 101, 105, 108).

Successful translation will be multidisciplinary. Radiation oncologists define the decision and dose context; geneticists and statisticians ensure valid discovery; radiobiologists test mechanisms; imaging and informatics specialists integrate exposure data; and patients identify outcomes that matter. Collaboration can begin during protocol design rather than after a variant is discovered. This alignment increases the chance that a study produces an interpretable result and a realistic next step (8, 9, 21, 22, 26, 27, 35, 101, 105, 108).

## 7 Conclusion

Radiogenomics has progressed from small candidate-gene studies toward GWAS, international meta-analysis, polygenic modeling, and functional investigation. An immunological synthesis clarifies how inherited susceptibility may act across the full course of normal-tissue injury: DNA and organelle damage initiate danger sensing; epithelial, endothelial, myeloid, and lymphoid cells shape inflammatory amplification and resolution; and persistent immune-stromal signaling contributes to senescence, vascular dysfunction, and fibrosis (4–7, 13, 41, 42, 85). The evidence is not uniform. General activation of an immune pathway supports biological plausibility, whereas clinical inference requires a replicated allele-to-endpoint association and variant-specific function in relevant normal tissue.

The path to translation is therefore immune-aware evidence integration rather than marker accumulation. Future studies should harmonize organ- and time-specific phenotypes, recruit multi-ancestry prospective cohorts, preserve detailed dose and treatment context, collect longitudinal immune measurements, and validate variants in allele-aware multicellular models. Integrated models may eventually support personalized surveillance, supportive care, and prevention trials, but no current common variant or polygenic score should independently reduce curative dose or target coverage. Common-variant scores, clinically interpreted rare variants, and imaging-derived tissue phenotypes should be evaluated as complementary components, while ICI-associated germline signals require validation in radiotherapy-exposed cohorts. Radiogenomics will become clinically meaningful when inherited susceptibility, immune mechanism, exposure, and a safe actionable decision converge (**Figure 6**).

**Figure 6:**
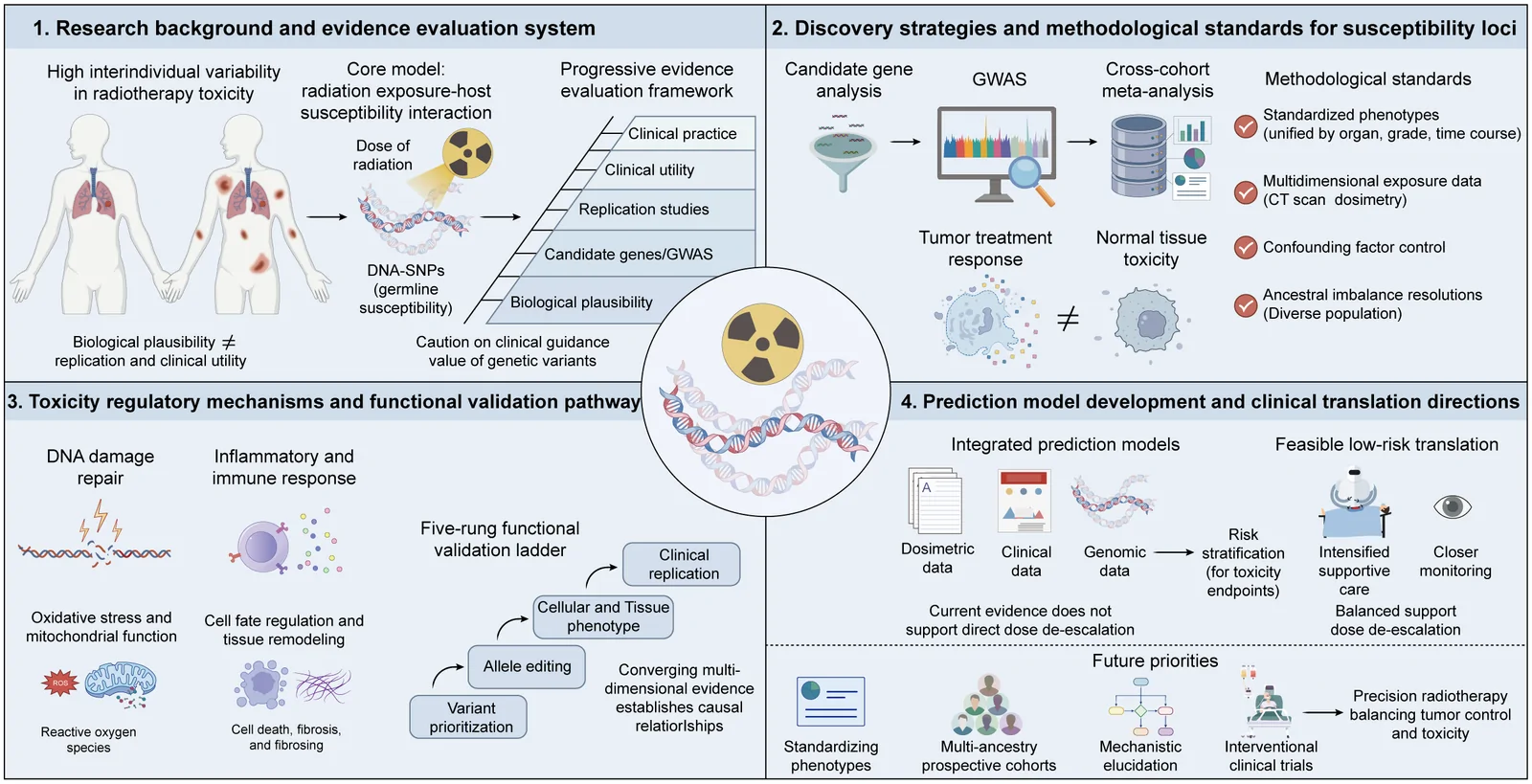
Roadmap for advancing immune and inflammatory radiogenomics of radiotherapy-induced normal-tissue toxicity from evidence evaluation and discovery standards to mechanistic validation, prediction modeling, and clinical translation.
